# A lumped parameter model of endoplasm flow in *Physarum polycephalum* explains migration and polarization-induced asymmetry during the onset of locomotion

**DOI:** 10.1371/journal.pone.0215622

**Published:** 2019-04-23

**Authors:** Christina Oettmeier, Hans-Günther Döbereiner

**Affiliations:** Institute for Biophysics, University of Bremen, Otto-Hahn-Allee 1, 28359 Bremen, Germany; Purdue University, UNITED STATES

## Abstract

The plasmodial slime mold *Physarum polycephalum* exhibits strong, periodic flow of cytoplasm through the veins of its network. In the special case of mesoplasmodia, a newly described starvation-induced, shape-constant morphotype, this periodic endoplasm streaming is the basis of locomotion. Furthermore, we presume that cytoplasm flow is also involved in signal transmission and signal processing. Mesoplasmodia motility resembles amoeboid locomotion. In contrast to other amoebae, however, mesoplasmodia move without extending pseudopods and retain a coherent, fan-shaped morphology throughout their steady locomotion. Attaining sizes of up to 2 mm^2^, mesoplasmodia are also much bigger than other amoebae. We characterize this particular type of locomotion and identify patterns of movement. By using the analogy between pulsatile fluid flow through a network of elastic tubes and electrical circuits, we build a lumped model that explains observed fluid flow patterns. Essentially, the mesoplasmodium acts as a low-pass filter, permitting only low-frequency oscillations to propagate from back to front. This frequency selection serves to optimize flow and reduces power dissipation. Furthermore, we introduce a distributed element into the lumped model to explain cell polarization during the onset of chemotaxis: Biochemical cues (internal or external) lead to a local softening of the actin cortex, which in turn causes an increased flow of cytoplasm into that area and, thus, a net forward movement. We conclude that the internal actin-enclosed vein network gives the slime mold a high measure of control over fluid transport, especially by softening or hardening, which in turn leads to polarization and net movement.

## Introduction

The acellular, multi-nucleated slime mold *P. polycephalum* can take on many shapes and sizes, depending on the mode of cultivation and various environmental parameters (e.g. nutrients, temperature, light). Typically, the slime mold forms large extended networks, characterized by a regular and vigorous flow of endoplasm (called shuttle streaming) through its veins. When placed in liquid shaking culture, shear forces tear the macroplasmodium apart and quasi-spherical, floating microplasmodia with diameters of a few hundred micrometers are produced. Regardless of shape and size, rhythmic oscillations of the cell periphery and the resulting flow of endoplasm are a characteristic feature of *P. polycephalum*. Cytoplasmic flow serves several purposes. First, it distributes nutrients, oxygen and cellular components throughout the cell body. Second, it is crucial for cell motility. In this work, we show that cytoplasmic flow is also a means of signal processing and distribution.

Rhythmic oscillations, caused by the contractile actomyosin cytoskeleton, are locally very well coordinated along the plasmodial body. They collectively and sequentially produce a pressure gradient, which induces protoplasmic streaming towards the leading edge (‘front’) of the plasmodium. In a stationary network, the flow through the network tubes is organized as a peristaltic wave, which is well-known [1] and has been elaborated on recently [2]. This peristaltic wave pattern leads to optimized transport. However, in migrating fragments of *P. polycephalum*, motility is achieved by the interplay of intracellular flow, adhesion, and traveling waves of contractile traction stresses [3, 4]. Intracellular pressure gradients, caused by the actomyosin system, form the basis for this type of movement. Another prerequisite for migration is the polarization of the cell into an anterior (front) and a posterior end (uroid). This can be achieved by the development of a chemical gradient, e.g. calcium, or by a softness gradient.

**Fig 1:**
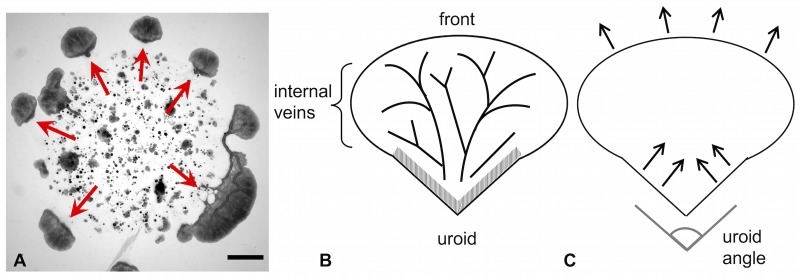
Mesoplasmodia migration pattern and schematic drawing. A) Mesoplasmodia emerging from microplasmodia plated on glucose-deficient agar. Image taken 7 hours after plating. Arrows indicate star-shaped migration pattern. Scale bar = 2 mm. B) and C) Schematic representation of a mesoplasmodium. B) The three most important regions involved in locomotion: the uroid (hatched area), internal veins, and front. An explanation is provided in the text. C) Proposed mechanism of the amoeboid locomotion employed by mesoplasmodia. Contractions are generated in the uroid, whose shape (uroid angle) influences locomotion speed. The front is pushed outwards passively by the flow.

Heeding internal and external cues, *P. polycephalum* can adapt and alter its shape and size. For an overview of how the network morphology is influenced by chemicals and substrate softness, see [5]. As we have shown before [6], microplasmodia can form networks via a percolation transition when placed on a 2-dimensional agar surface. However, under starvation conditions, this transition does not occur. Instead, several disconnected, autonomous, millimeter-sized units form and migrate outward from the site of inoculation [7] (see Fig 1A).

This new morphotype, which we termed mesoplasmodium (because its size places it between the micrometer-sized microplasmodium and the large macroplasmodium), is the focus of the present study. We establish it as a model system, because mesoplasmodia have a stable and defined morphology (for a schematic, see Fig 1B), a persistent shape, and move on straight trajectories for hours before they become stationary and once more transition into networks (a sketch of the locomotion pattern can be found in Fig 1C). Their ultrastructure has recently been described by us [8]. The uroid (hatched area in Fig 1B) contains, apart from the ubiquitous cortex, specialized actin fibres which enable vigorous contractions. The uroid is morphologically different from the front. Internal veins transport flowing cytoplasm (endoplasm) through the more stationary ectoplasm. The veins have flexible boundaries and thus, pressure waves arrive at the front in an altered way, with a different frequency spectrum. The front lacks the organized actin fibres of the uroid and is thus softer than the back. As we have described earlier [8], blebbing at the front, an asymmetrical actin distribution throughout the cell, and the presence of actin asters in the uroid, but their absence in the frontal area, provide a strong argument for a softer front. Furthermore, as Lewis and coworkers have shown [9], a softer front is a prerequisite for cell migration. In order to move forward, the cell needs to polarize and then establish a softness gradient. We were able to demonstrate this exact mechanism with our circuit model. If a symmetrical AC circuit, which is the equivalent of an unpolarized cell, suddenly becomes softer in one place (which is manifested as a local increase of electrical capacitance), there is a net current (i.e., the equivalent of net volumetric flow of endoplasm) which streams towards the softer area. It pushes the soft frontal membrane outwards. At the same time, the uroid keeps contracting rhythmically. Since the cytoplasm volume does not change, it follows that the bulk of cytoplasm is shifted towards the front, creating movement. As for a softening of the frontal actomyosin cortex during locomotion, this has been experimentally shown for *Dictyostelium discoideum*, a closely related cellular slime mold [10]. Chemotaxis is a very prominent and important process in *P. polycephalum*. It is known that chemotactic receptors on the cell surface interact directly with the cytoskeleton in *D. discoideum* [11], affecting for example actin polymerization and pseudopod extension. In *P. polycephalum*, the nature of the chemotactic receptor(s) is still unknown, but it is very likely that the mechanism is similar.

*P. polycephalum*, like every other living organism, perceives its environment and reacts to it. In other terms, the plasmodium can be described as a cellular processor. The input variables are optical, chemical or mechanical cues. The slime mold processes this input, and, depending on the task at hand, creates an observable output. The output which is easiest to observe is mechanical in nature, i.e. shape, position and oscillations of the slime mold. Investigating how *P. polycephalum* processes and distributes information sheds light on basic emergent processes that lead to complex behavior in the absence of neuronal structures. We propose that the morphology and the physical properties of the slime mold and its (non-neuronal) information processing capacities are tightly interconnected. Computational processes are based on physically grounded dynamics (such as electrons moving through a circuit, or action potentials on neurons). As opposed to organisms with a brain as central processing and controlling unit, in the slime mold this ‘brain’ is its morphology. There is no particular location where memory or sensory input processing can be found; it is distributed throughout the entire body. The hydrodynamic network of *P. polycephalum* can thus be said to possess information processing capabilities. The oscillatory flow of cytoplasm is responsible for locomotion and signal processing, with the encoding of information being achieved by, e.g., frequency modulation.

After detailing the observed motility patterns, we use a hydrodynamic combined lumped-distributed model to describe and reproduce the cytoplasm flow of *P. polycephalum*. The mechanism underlying the movement process of *P. polycephalum* is likened to the operation of an equivalent electrical circuit consisting of two basic passive electrical elements: resistor and capacitor. These models are usually lumped, meaning the equivalent circuits do not contain spatial information, which we aim to include in our model. Within the slime mold, a complex multitude of chemical and physical processes interacts to produce behavior, but the present analogy may nevertheless be very useful in better understanding the locomotion mechanism and signal processing capabilities of the slime mold.

## Materials and methods

### Cell culture and mesoplasmodium production

We used the strain WT31 [12]x LU898 [13] which was kindly provided by W. Marwan. Microplasmodia were grown in liquid shaking culture as described in [14]. After 6 days, the slime molds have depleted the medium of glucose, which was confirmed by a glucose monitoring strip test (Combur-Test, Roche). The microplasmodia were then taken out of the liquid culture and plated onto an agar plate containing glucose-deficient SDM-agar (prepared after [15]). Once on the agar surface, microplasmodia form aggregates and fuse. At ∼ 3 hours after plating, small plasmodia begin to leave the boundaries of the patch and move outwards in a star-shaped fashion. We call those autonomous, steadily migrating units mesoplasmodia. After approximately 10 hours, mesoplasmodia cease their straight, outward migration and transition into stationary networks in a topological transition creating numerous holes and handles. Directional persistence is evident for approximately 7 hours. All mesoplasmodia were observed and recorded within this seven-hour interval.

### Image acquisition

Light microscopy of migrating mesoplasmodia was performed using a Zeiss Axiovert 25 equipped with a 5x Zeiss CP-Achromat with a numerical aperture of 0.12. For higher-resolution imaging of internal flow, a Zeiss Axio Oberver.Z1 was used. Bright field imaging was performed with a Zeiss Achro Plan 10x (numerical aperture 0.25). All experiments were carried out at room temperature.

### Analysis of leading edge velocity

**Fig 2:**
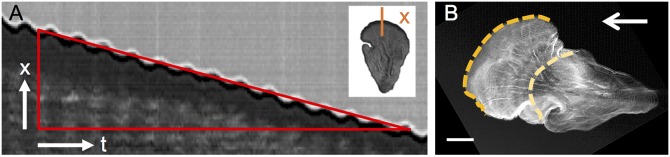
Kymograph. A) Kymograph of the growth front of a mesoplasmodium. x = spatial dimension, t = time. Inset: single frame of a time sequence. Kymograph is taken along orange line. B) Output image of a time series (standard deviation). Yellow dashed line = first frame of time series, orange dashed line = last frame. Scale bar = 20 μm. Arrow denotes direction of migration.

The analysis of the average leading edge velocity of mesoplasmodia was performed with the open-source software FIJI [16] and the Kymograph plugin written by J. Rietdorf and A. Seitz. Kymographs (schematic see Fig 2) with a width of 1 pixel were generated on image sequences (stacks) of moving mesoplasmodia. Kymographs of the output stacks of optical flow analysis were also used to obtain flow velocities along internal veins.

### Optical flow analysis

We used the Horn-Schunck method of estimating optical flow [17]. A detailed description of the method can be found in S1 Text.

### Time series analysis

Two-dimensional representations of time series were created using standard deviation in the z-dimension (time), resulting in a single output image for each image stack. The standard deviation of all images in a stack was calculated in FIJI. Each of the output image’s pixels contains the standard deviation over all images in the stack at the particular pixel location. Areas with strongly fluctuating flow (high standard deviation) thus become visible and appear white, whereas areas with little flow variation have a low standard deviation and appear dark.

### Contour detection

The detection of mesoplasmodium contours, based on the principles of active contour or ‘snake’ algorithms [18], was implemented in MATLAB2015b (The Mathworks) using custom-written routines by E. Bernitt [19].

## Results and discussion

### Directional persistence and cell speed

**Fig 3:**
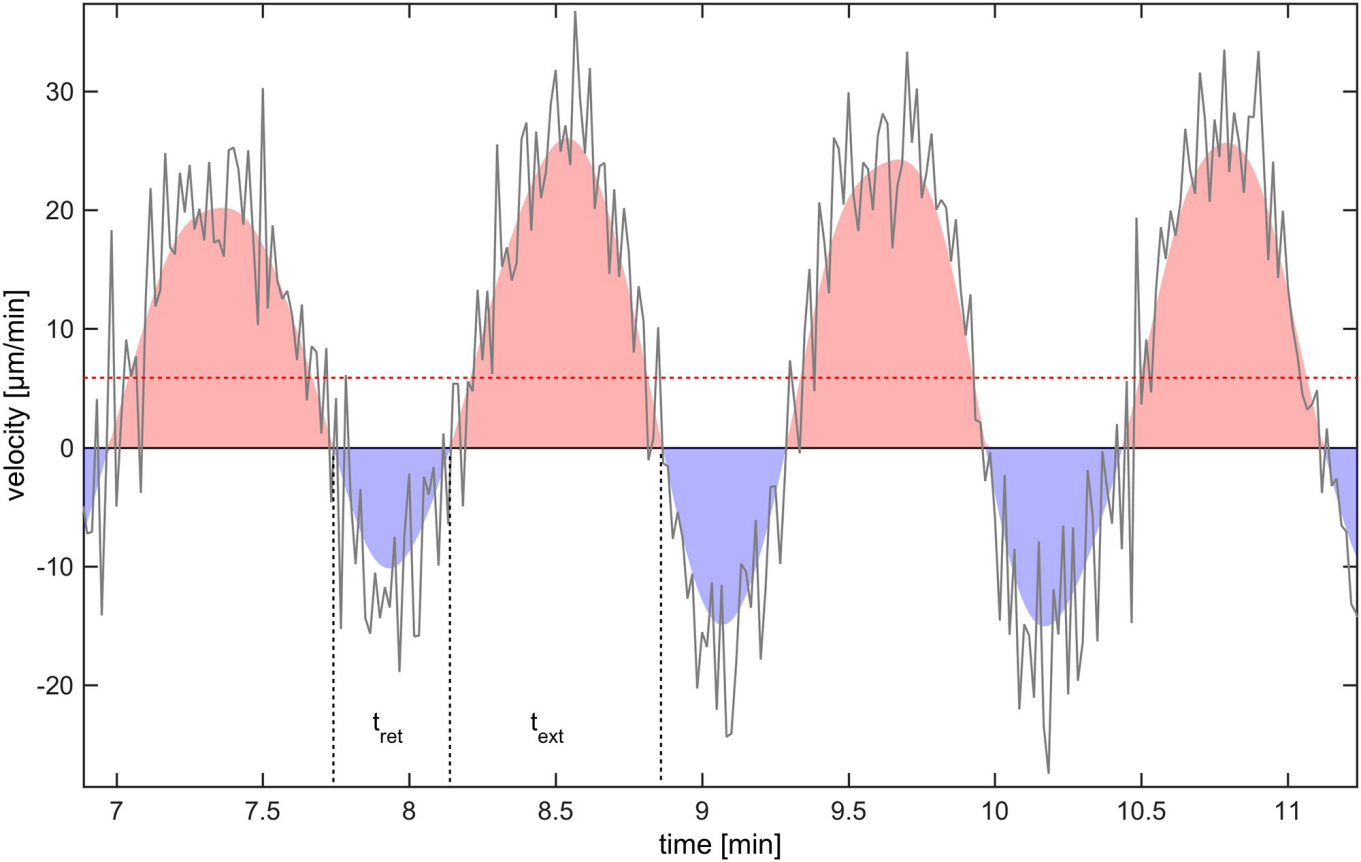
Movement speed of frontal membrane. Red dashed line = average velocity. Negative values = movement of cell contour towards center of mass. *t*_*ret*_ = membrane retraction time, *t*_*ext*_ = membrane extension time.

We followed mesoplasmodia for several hours and analyzed their motion with respect to velocity, area, and shape (circularity *f*_*circ*_) over time. These locomotion parameters are given for six mesoplasmodia in S1 Table. We found that mesoplasmodia migrate at constant speeds for up to 7 hours and maintain straight trajectories away from the original patch (see Fig 1A and S1 Fig). For unicellular eukaryotic cells, such behaviour is very uncommon. Usually, amoeba such as the cellular slime mold *D. discoideum* show directional persistence times of only ∼10 minutes in the absence of a stimulus [20]. We measured migration speed during this phase of directional persistence as the velocity of the leading edge in direction of movement, which was obtained from kymographs (see Fig 2A). Mesoplasmodia travelled with an average of 6 μm/min to 17 μm/min. Cell speeds show characteristic oscillations around their average values (see Fig 3).

Although a single mesoplasmodium maintains its shape during migration, mesoplasmodia differ in overall shape and size from each other. The general form is always the same, see Fig 1, in that a front and a tail region are clearly distinguishable. Thus, even from looking at a single still frame, the direction of movement is always discernible. Independent of size, the tail region can be elongated (acute uroid angle) or oriented almost parallel to the front (obtuse uroid angle). Uroid angles for six mesoplasmodia can be found in S1 Table.

Mesoplasmodial locomotion is characterized by a very long directional persistence and a constant average cell speed, which fluctuates in a sinusoidal pattern. The mesoplasmodium achieves net forward movement by a long frontal membrane extension time (*t*_*ext*_) and a short retraction time (*t*_*ret*_), see Fig 3. Extension time is the time over which the frontal membrane is expanded in direction of movement (calculated for an entire time series), and retraction time is the time span over which the membrane moves back towards the contour center. For all examined mesoplasmodia, the ratio of membrane extension to retraction time lies between 1.4 and 2.3. The overall area of a migrating mesoplasmodium oscillates regularly, with average periods of *T*_*area*_ = 1.20 min. This parameter seems to be independent of size. The cell shape of migrating mesoplasmodia was assessed by the circularity factor *f*_*circ*_ which takes into account area *A* and perimeter *P*.

$$\begin{array}{c} f_{circ}=\frac{4\cdot \pi \cdot A}{P^{2}} \end{array}$$ (1)

The circularity *f*_*circ*_ of a perfect circle is 1, whereas elongated cells assume values of <1. We found typical values of *f*_*circ*_ to lie between 0.84 and 0.92. The shape remains almost constant during migration (S2 Fig), with regular fluctuations. Phases of high circularity roughly coincide with periods of slow locomotion (S3 Fig).

### Cell shape dynamics

**Fig 4:**
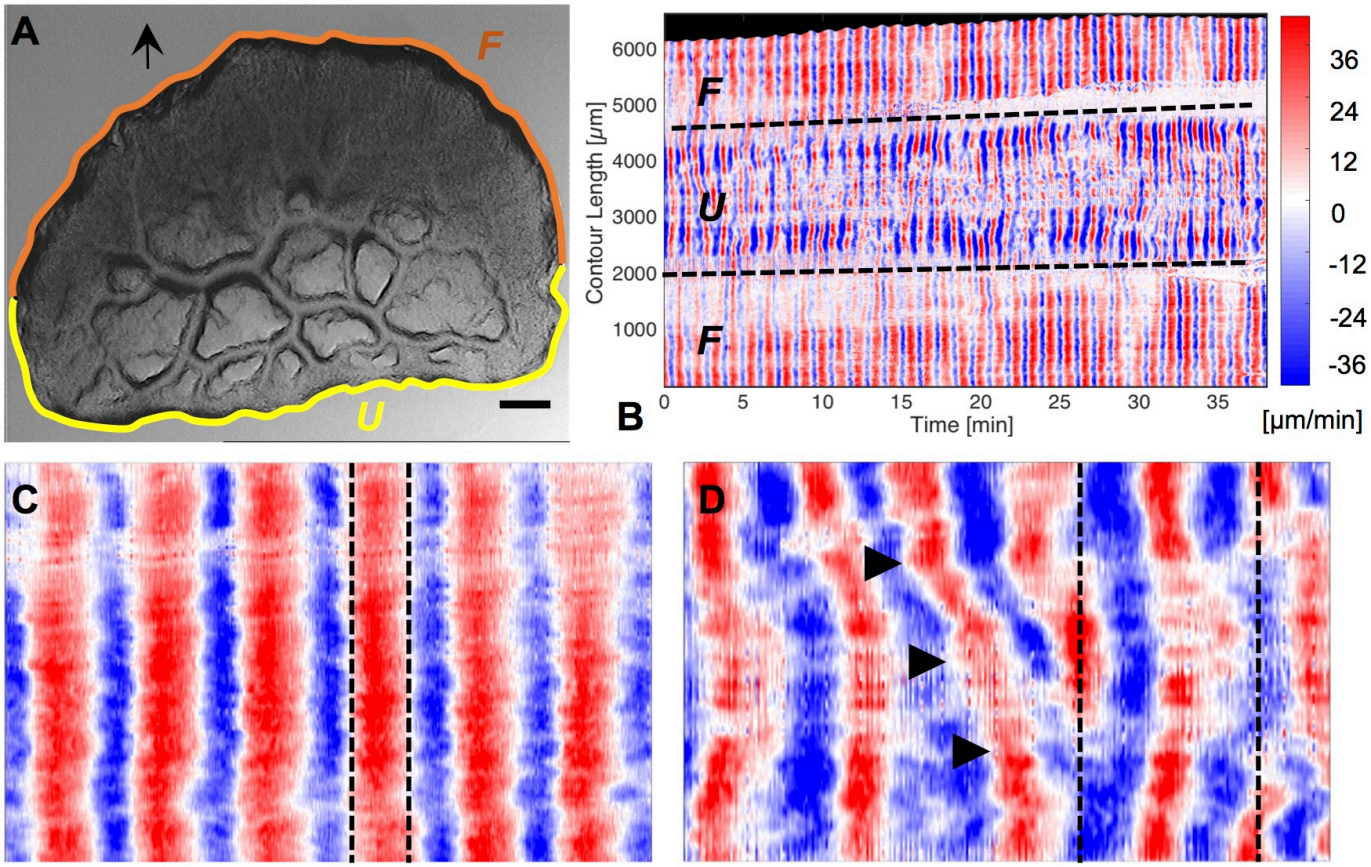
Contour dynamics. A) Still frame taken from a 40 minute recording of a mesoplasmodium with an obtuse angle. Scale bar = 200 μm. Arrow denotes the direction of movement. U = uroid, F = front. White arrowhead correponds to contour length 0 resp. 6000 μm in panel B. B) Velocity chart of same mesoplasmodium. Data was obtained as given in section Contour detection. Dashed lines mark points on the contour where the uroid (U) transitions into the front (F). C) Magnified detail of the front shows uniform membrane extrusion and retraction. D) Magnified detail of uroid region shows simultaneous movements in opposite directions (dashed lines) and lateral wave phenomena (arrowheads).

So far, we have described the overall movement pattern and shape of mesoplasmodia. Next, we will investigate the dynamics of locomotion. Velocity charts reveal different patterns of membrane extension and retraction in the frontal zone and the uroid. Fig 4 shows the contour dynamics of an exemplary mesoplasmodium. As per our definiton, outward movement of the contours has a positive sign and is given the colour red in the velocity chart. Movement towards the contour center has a negative sign and is represented by blue colour. The oscillations of a mesoplasmodium are not homogenous along its contour, with the exception of the front. The frontal periphery expands and retracts evenly over its length (see Fig 4C). The uroid, however, shows a more versatile oscillation pattern (see Fig 4D). The occurence of tilted lines of equal velocity indicates lateral waves moving along the contour (see Fig 4D). For microplasmodia, similar phenomena, i.e. lateral waves along the membrane and standing wave patterns, have been described by us [14]. Lateral waves also occur in other cell types, for example in T cells, mouse fibroblasts and *Drosophila* wing disk cells [21]. The appearance of a common spatiotemporal pattern of membrane movement in a variety of cell types suggests an underlying universal pattern, most likely associated with actin.

**Fig 5:**
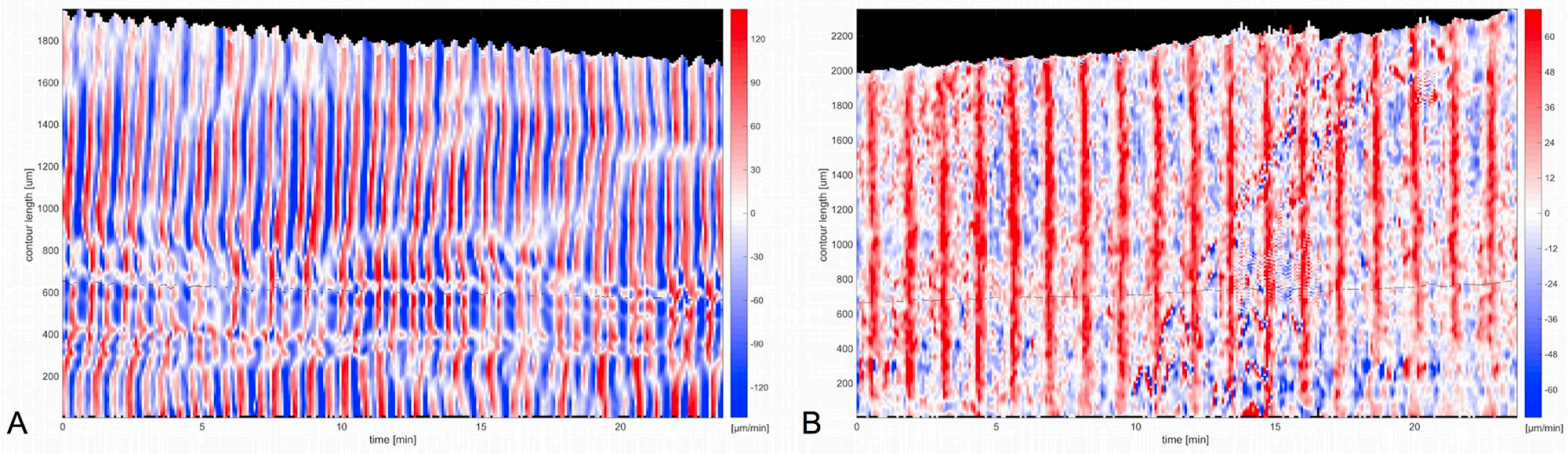
Frequency selection. A) Velocity chart of uroid of mesoplasmodium. B) Velocity chart of front. Data was obtained as given in section Contour detection.

The front is also distinguished from the uroid by different oscillation frequencies. The back of a mesoplasmodium usually shows a higher frequency of oscillation than the front, see Fig 5. Here, the back (Fig 5A) oscillates with a period of 1.20 min, and the front (Fig 5B) oscillates with a period of 2.40 min. Furthermore, the membrane velocities are higher in the back. A video of this mesoplasmodium can be found in S1 Movie.

### Internal flow patterns

The analysis of the cell periphery shows very different oscillation patterns between front and uroid. Pressure differences caused by uroidal contractions lead to a propagation of waves from the back towards the front. We investigated the internal flow of endoplasm throughout the mesoplasmodium by means of optical flow analysis. Every examined mesoplasmodium showed the patterns described below, albeit at different levels of distinction. For the sake of clarity, we therefore chose to present data which best highlighted the observed behavior.

**Fig 6:**
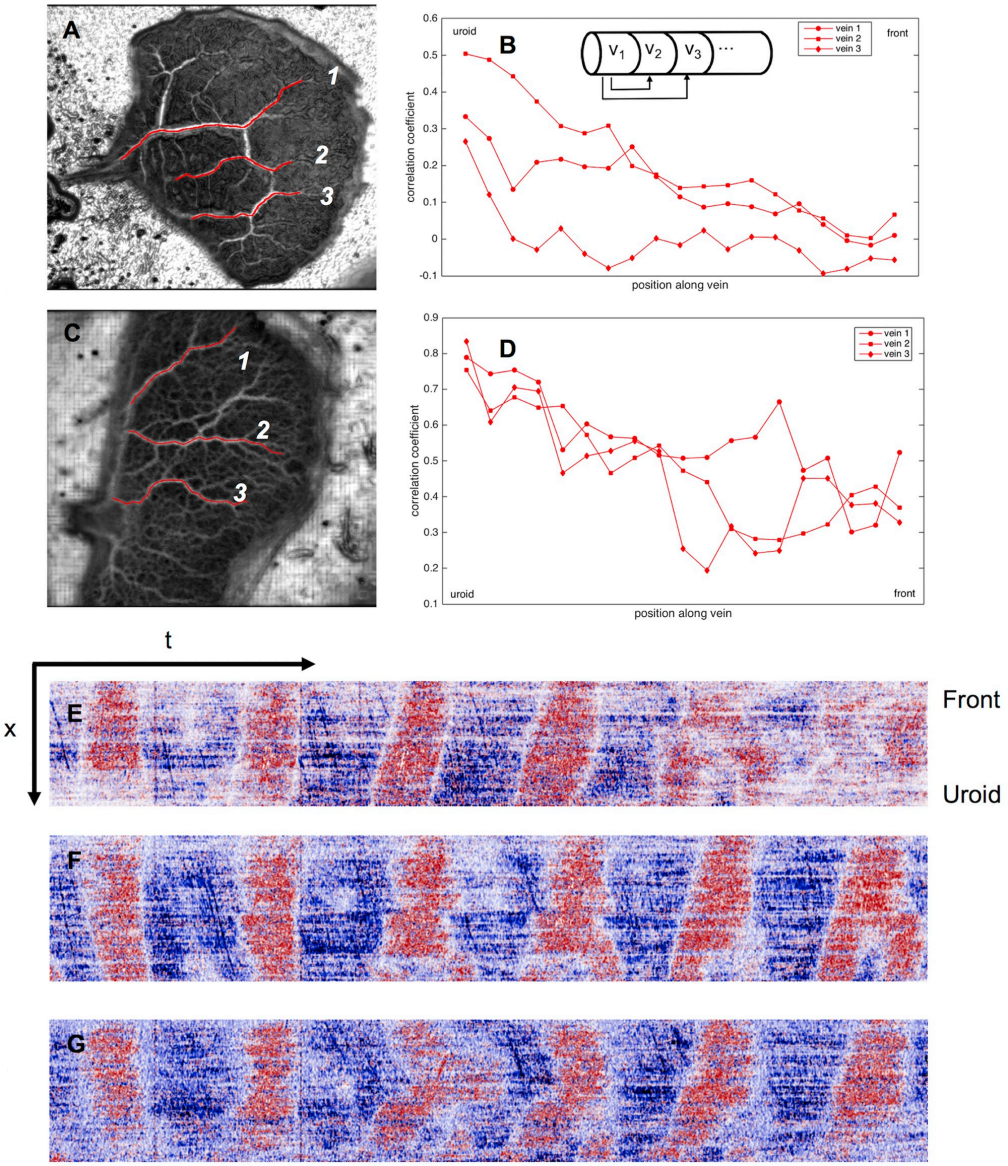
Flow pattern along veins. A) Satellite with acute uroid angle (time series over 7 minutes). Veins (red) run from uroid to front. B) Cross-correlation of flow velocities along the three veins. The velocity profile of the vein segment closest to the uroid is correlated with that of every subsequent segment along the vein (see inset). The correlation decreases slightly from back to front. C) and D) Similar analysis for satellite with obtuse angle. Time series over 14.5 minutes. E-G) Kymographs (see method section Analysis of leading edge velocity) taken along three veins of the mesoplasmodium in C), denoted with numbers 4-6. x = spatial dimension (each vein is ∼ 800 μm long), t = time (∼ 15 min). Bottom edge of each kymograph corresponds to vein area close to uroid; top edge is closest to the front. Along each vein, the transition from uroidal to frontal oscillation pattern (frequency selection) can be observed: The oscillation frequency is almost twice as high closest to the uroid as near the front.

From the evaluation of time series (see section Time series analysis), it becomes evident that at least major veins are persistent over longer periods of time (up to hours) while the satellite moves forwards (see Fig 6A and 6C). Thus, the majority of veins are stationary in regard to the agar surface. In the frontal area, flow channels are more ephemeral. The veins are deconstructed as the uroid retracts, and newly formed at the expanding front. There are usually only two or three dominant veins, running parallel to the longitudinal axis, which are responsible for the bulk of the flow (veins traced in red in Fig 6A and 6C).

**Fig 7:**
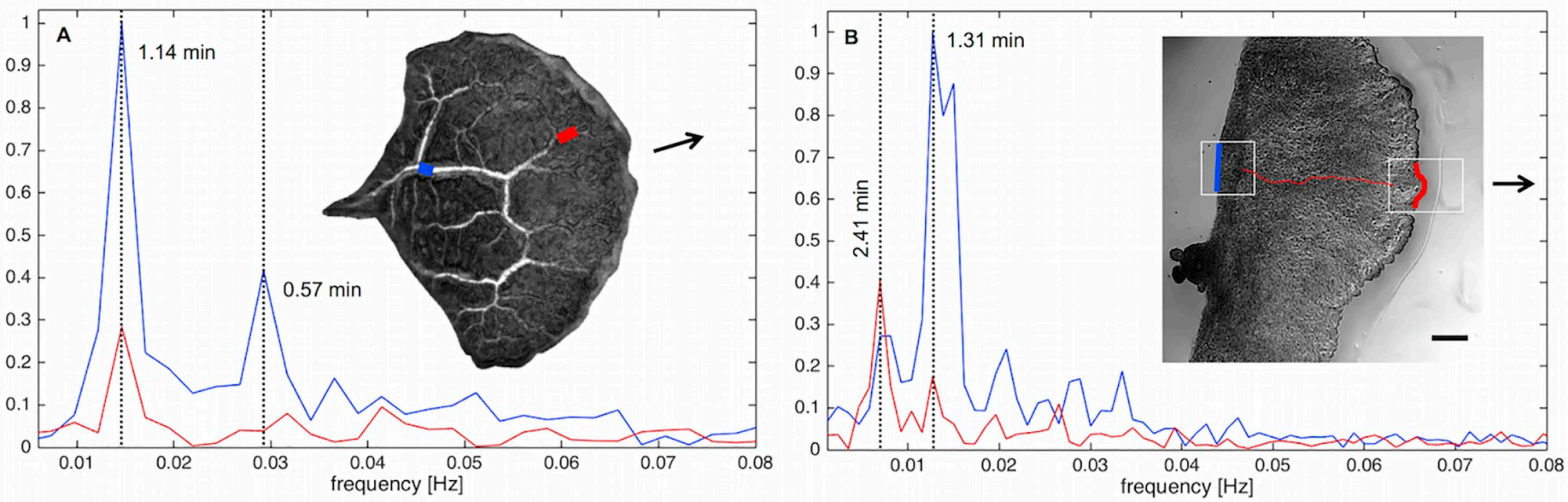
Fast Fourier transform (FFT) of mesoplasmodial oscillations. Examples of changing frequencies of endoplasm flow throughout a mesoplasmodium (A) and differences in the oscillation pattern between frontal and uroidal membrane (B). Arrows denote direction of movement. A) Data obtained from optical flow analysis. Blue denotes a segment in the back of the mesoplasmodium, red is a segment closer to the front. Whereas a frequency with a period of 1.14 min can be detected everywhere along the length of the vein (although getting less pronounced further away from the uroid), the higher frequency also present in the uroid (0.57 min) is filtered out towards the front. The mesoplasmodium as a whole shows area oscillations with a period of 0.62 min. B) Red = oscillations of the frontal membrane. Blue = oscillations of the uroidal membrane (see white boxes). Data obtained from kymographs.

Flow within the internal veins of a mesoplasmodium has a sinusoidal pattern, mirroring the extension-retraction pattern of the uroid’s periphery. Performing cross-correlations of the velocity profiles throughout a vein from beginning (uroid) to end (front) show a gradually declining correlation coefficient (Fig 6B and 6D). This means that segments which are in close proximity to each other tend to have more similar flow patterns than segments that are further distant from each other. The self-similarity of the wave decreases along the longitudinal axis. Typical flow velocities in internal channels ranged from 5 to 100 μm/min, with highest flow velocities occurring in the middle of the mesoplasmodium. A remarkable phenomenon that originates in the uroid and propagates through the veins from back to front is a change in oscillation frequency. The resulting pattern (both high and low frequencies in the back, only low frequencies in the front) resembles an electronic low-pass filter. By performing a fast Fourier transform (FFT) on the oscillations, we obtained the dominant frequencies. Fig 7 shows different frequency spectra of uroid and frontal region. Frequency spectra for different regions of a dominant vein can be seen in Fig 7A. Segments closer to the uroid (blue) have a higher oscillation frequency that is absent in segments closer to the front (red). Whereas low frequencies are present in both uroid and front, higher frequencies are missing in the front. We show that, from back to front, a frequency selection takes place. Fig 7B shows the comparison of frequencies present in the uroidal membrane (blue line) and the front (red line). The observed frequencies differ between individuals, but the mechanism of frequency selection is the same.

To summarize this observation, we found that the oscillations of the uroid (periphery as well as internal flow) contain both high and low frequency components. As endoplasm flows towards the front, higher frequencies are filtered out along the way.

### Lumped model of cytoplasm flow

We aim to provide a predictive model useful for the explanation of dynamic phenomena in slime mold. To this end, we develop a lumped parameter approach that allows us to analyze the behavior of mesoplasmodia with modest computational effort. Certain concepts in electrical circuits bear a strong similarity to fluid flow in networks of compliant tubes. Therefore, we can derive fundamental equations by applying electric circuit theory. Voltage in an electric system corresponds to pressure in a fluidic system, current to volumetric flow rate. In both fluidic and electric circuits, resistors and capacitors affect the flow of electrical current. Fluidic resistance is due to internal friction within the fluid. The fluidic equivalent to a capacitor lies in the elastic properties of the tube wall: An increase in pressure causes the elastic vein to expand and store fluid which it then releases, much like a capacitor stores and releases electric charge. The lumped parameter concept provides a tool to examine the dynamics of pulsatile flow in a mesoplasmodium as a whole. This modeling approach has been used extensively in hemodynamics [22, 23].

The slime mold’s flow of cytoplasm follows complex patterns in space and time. At the core of the ubiquitous relaxation-contraction cycles are the interactions between actin and myosin in the cortical cytoskeleton. However, the underlying primary (or biochemical) oscillator is still unknown. It may be founded in biochemical pathways [24], but could also be based on mechanochemical processes and / or mechanical interactions between different regions [25]. We propose a model in which observed patterns are, in great parts, due to hydrodynamic processes without the need for biochemical signals. In the following, we will detail our lumped model and focus on the observed frequency selection phenomenon.

However, a challenge for the construction of a lumped model is that many parameters like cytoplasm density, viscosity and elasticity have to be known. Channel dimensions can be taken from microscopic images [8], but rheological data for *P. polycephalum* are scarce [26].

#### Reynolds number

To estimate what kind of flow regime is predominant in mesoplasmodia, we calculated the Reynolds number *Re* (Eq 2)

$$\begin{array}{c} Re=\frac{\rho_{cyto}\cdot v\cdot d}{\eta } \end{array}$$ (2)

where *ρ*_*cyto*_ is the cytoplasm density, *v* the average flow velocity, *d* the typical diameter of a flow channel, and *η* the viscosity. The values we used for the calculations of Reynolds and Womersley numbers are given in Table 1. Average flow velocity shows some variation, depending on morphotype and position within the cell body. The mesoplasmodia which were the subject of this study showed, on average, a much lower flow speed, especially in the tiny flow channels in the frontal region. Flow through the large and well developed veins of macroplasmodia can reach up to 60000 μm min^−1^, but this flow differs very much from the situation in mesoplasmodia, which are characterized by a much smaller cytoplasm volume, smaller size, and ultrastructure of the flow channels. Very often, the flow channels are simply constituted of faster-flowing endoplasm which is pushed through the more stationary cytoplasm surrounding it. This explains the much lower flow velocities. The calculated Reynolds number (2.37 × 10^−8^ − 4.75 × 10^−8^), depending on flow velocity, is very low as compared to values at which flow is turbulent, thus we can assume smooth laminar flow which is dominated by viscosity. Therefore, endoplasmatic flow may be described by the equation for Poiseuille flow in a tube and, most importantly, inertial effects are negligible. In our considerations, we regard the endoplasm as an incompressible fluid, i.e., we take the density as a constant. However, cytoplasm is a non-Newtonian fluid.

**Table 1:** Parameters used for the calculation of *Re* and *α*.

*ρ*_*cyto*_	1120	$\frac{kg}{m^{3}}$	[27]
*v*	5–100	μm min^−1^	
*d*	70	μm	
*η*	0.275	$\frac{N\cdot s}{m^{2}}$	[28]
*a*	20	μm	

#### Womersley number

The Womersley number *α*, which is dependent on the angular oscillation frequency *ω*, can be used to determine the flow profile. This dimensionless expression relates pulsatile flow frequency to viscous effects. In a typical case for mesoplasmodia (*ω* = 0.08 rad/s and radius *a* = 20 μm), the Womersley number *α* becomes

$$\alpha =a{\left(\frac{\omega \rho_{cyto}}{\eta }\right)}^{\frac{1}{2}}=0.00036$$ (3)

0.08 rad/s corresponds to an oscillation frequency of 0.013 Hz, which means a period of 1.31 min. For *α* ≲ 2, viscous forces tend to dominate the flow, and velocity profiles are parabolic in shape. The oscillations of the slime mold which are relevant to flow are well below 1 Hz, usually in the range of 0.007 to 0.02 Hz.

#### Model of an internal vein segment

**Fig 8:**
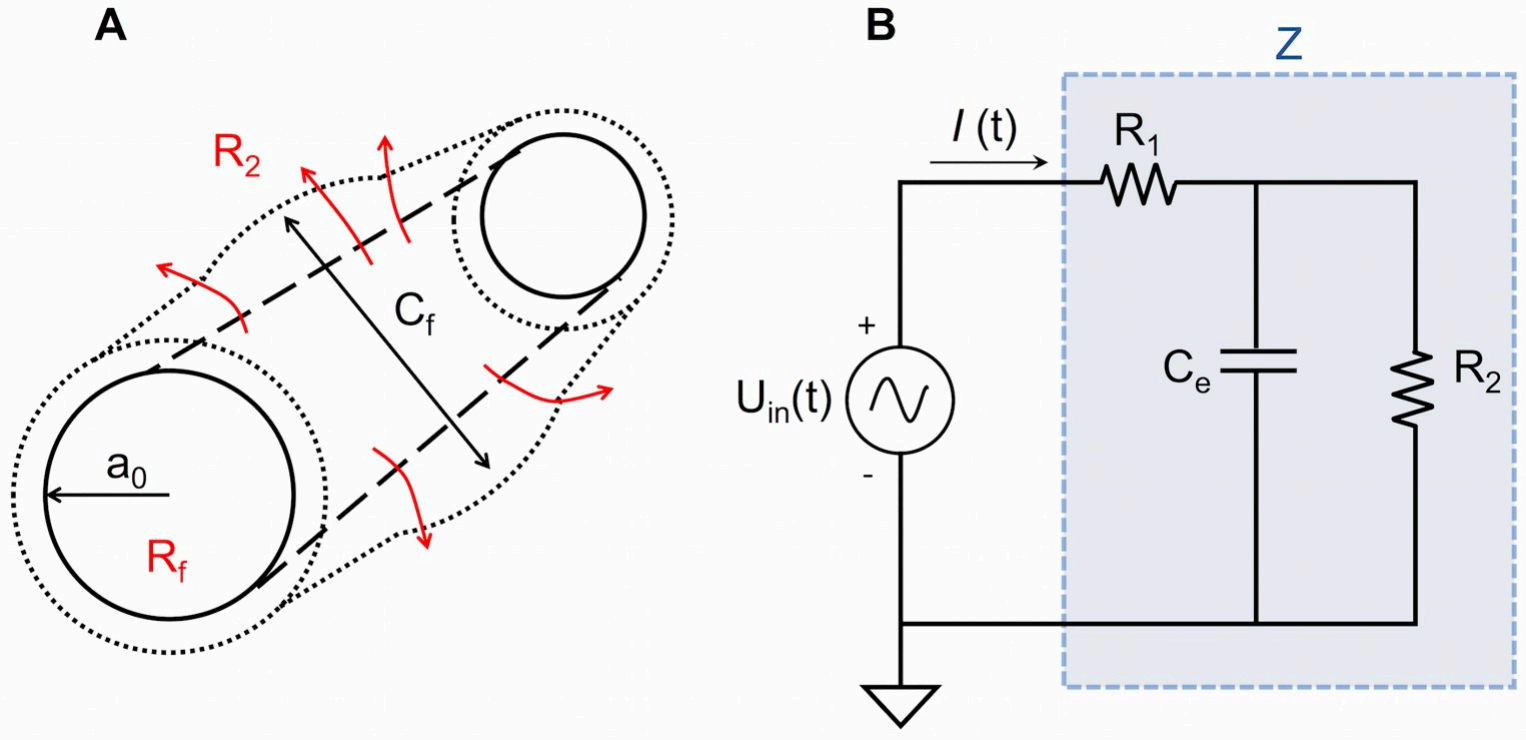
Schematic of a three-element Windkessel equivalent circuit. A) Schematic drawing of a tube segment of an internal vein. *a*_0_ = radius, *C*_*f*_ = fluidic capacitance, *R*_*f*_ = fluidic resistance. *R*_2_ = leakage due to permeable ‘walls’. B) Schematic drawing of RC circuit. The blue box (denoted with Z) represents the impedance of one singele 3-element Windkessel (see Fig 9). *U*_*in*_(*t*) = input voltage signal, *R*_1_ = resistance due to internal friction of the cytoplasm, *R*_2_ = resistance due to leakage through vessel wall, *C*_*e*_ = electric capacitor, *I* = current.

The internal flow network of a mesoplasmodium is a fluidic network with deformable features. Each segment is a tube with a radius *a*_0_ Fig 8 and, theoretically, elastic walls.

However, a peculiarity of mesoplasmodial internal veins is that they do not possess membranes to separate them from the surrounding ectoplasm [8]. Rather, channels are created by pressure-driven flow [29]. In our model, we consider the vessels as compliant and able to expand in response to an inflow of endoplasm (see Fig 8A). Given the lack of a solid tube wall, they are also permeable. The lack of substantial walls and the permeability create a similar situation as in capillaries and venules in the blood circulatory system. Cytoplasm constantly leaves and enters the flow channel. This is denoted by *R*_2_, an extra resistance added to the circuit to account for leakage through the sides of the vessel [30].

The circuit described above and depicted in Fig 8B is known as a three-element Windkessel model and is widely used, especially in hemodynamics, to model e.g. coronary blood flow. The Windkessel effect helps in damping the fluctuation in fluid pressure during an oscillatory input. Fluctuations in pressure are attenuated or dampened, and the fluid flow becomes more constant. The basic relations of the Windkessel model can be derived from Navier-Stokes equations [31]. We decided on the three-element Windkessel, because it provides one resistance to cytoplasm flow and a second one to simulate the permeability of flow channels. Thus, it represents the most important (experimentally accessible) parameters: resistance to flow, compliance of vessel ‘walls’, and loss of flow due to permeability. The four-element Windkessel includes an inductor. However, we ruled out inertance because the slime mold oscillates at very low frequencies.

**Fig 9:**
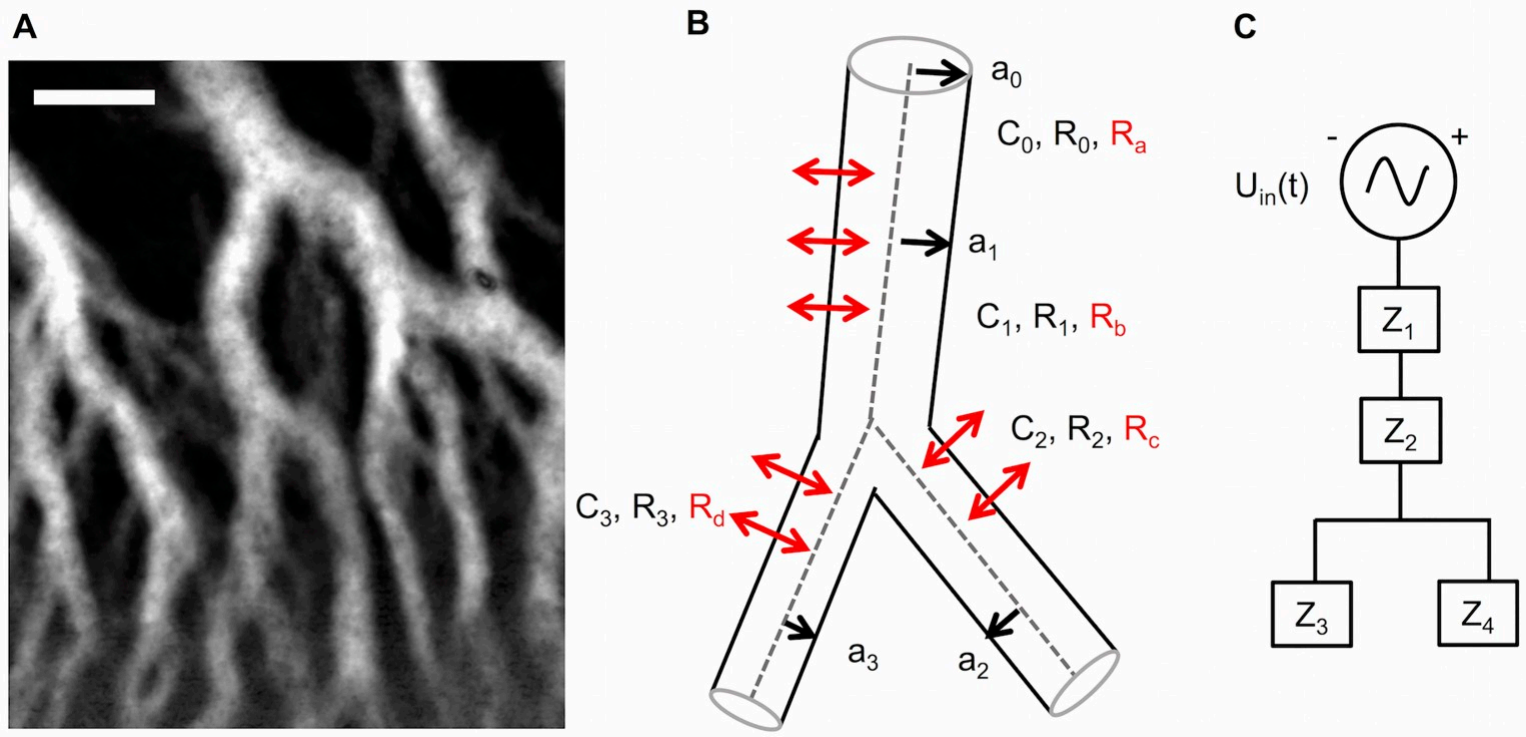
Modeling internal veins as an equivalent electrical circuit. A) Time series (standard deviation) of a mesoplasmodial internal vein network. Scale bar = 50 μm. B) Model of branching vein. Each branch has its own characteristic length, radius and resulting fluidic resistance and capacitance. C) The branching vein, drawn as an electrical circuit. It consists of 4 single 3-element Windkessels (Z1—Z4).

The internal veins within a mesoplasmodium form a flow network of interconnected and branching pipes (see Fig 9A) through which endoplasm flows in a pulsatile flow pattern. Each of the more complicated branching tubes can be modeled as a different arrangement of simpler segments (see Fig 9B and 9C). Each letter Z in Fig 9C stands for the impedance of a single three-element Windkessel, i.e., each Z is a combination of two resistors and a capacitor. This representation highlights one of the advantages of the lumped model: For each Z, a circuit with individual elements can be employed and the parameters can be set according to measured data, for example radius and tube length.

We chose the configuration of Fig 9C for our branching vein model because it corresponds to the typical conditions within a mesoplasmodium: The veins branch only in the final third of their length, towards the front. This is why we assign two 3-element Windkessels to the ‘stem’ of the Y and one 3-element Windkessel to each ‘arm’.

#### Fluidic resistance

The resistance R for flow in a tube can be expressed in terms of properties of fluid (viscosity *η*) and tube (length *l*, radius *a*):

$$\begin{array}{c} R_{f}=\frac{8\eta l}{\pi a^{4}} \end{array}$$ (4)

#### Fluidic capacitance

Fluidic capacitance *C*_*f*_ represents the compliance of a tube, i.e., the elasticity of the channel ‘walls’. Capacitance is equal to the change in volume divided by the change in pressure.

$$\begin{array}{c} C_{f}=\frac{\Delta V}{\Delta p} \end{array}$$ (5)

We follow the argumentation by Fibich and coworkers [32], who constructed a model for blood flow in coronary capillaries. Capillary vessels in the human body can be compared to the internal flow channels in *P. polycephalum* because they share certain characteristics: Their length is comparable (∼ 1 mm), and they have very thin and permeable walls. In a previous study [33], it was demonstrated that pressure changes in capillaries are linearly related to the strain, so that

$$\begin{array}{c} \Delta p=E\cdot \varepsilon \end{array}$$ (6)

where *E* is Young’s modulus; and *ε* is the strain measure defined as

$$\begin{array}{c} \varepsilon =\frac{1}{2}[{\left(\frac{a(z,t)}{a_{0}}\right)}^{2}-1] \end{array}$$ (7)

*a*(*z*, *t*) is the flow channel radius, and *a*_0_ is the reference radius under zero transmural pressure. Fibich and coworkers define a normalized radius *h* as

$$\begin{array}{c} h\left(z,t\right)=\frac{a(z,t)}{a_{0}} \end{array}$$ (8)

Inserting *h* into Eq 7, and from Eq 6 it follows that *h*^2^ can be regarded as a pressure:

$$\begin{array}{c} h^{2}=1+\frac{2\Delta p}{E} \end{array}$$ (9)

The equation for the compliance

$$\begin{array}{c} C_{f}=\frac{\partial V}{\partial P}=\frac{\partial }{\partial P}\left(\pi {a_{0}}^{2}l\right) \end{array}$$ (10)

reduces, with regard to Eqs 8 and 9, to

$$\begin{array}{c} C_{f}=\frac{2\pi {a_{0}}^{2}l}{E} \end{array}$$ (11)

with *E* being Young’s modulus, and *a* and *l* radius and length of the segment, respectively. The magnitude of *C*_*f*_ strongly depends on Young’s modulus *E*. In our investigation of the ultrastructure of mesoplasmodia [8], we have shown that the internal veins of mesoplasmodia (and also within the sheet-like growth fronts of macroplasmodia) are permeable and lack membranes. They are, however, surrounded by a regular F-actin meshwork. The calculated capacitance strongly relies upon the elastic modulus of the vessel ‘wall’, as can be seen in Eq 11. We measured the Young’s modulus of whole microplasmodia by indentation and found it to be in the range of 16.4 kPa [26]. However, this is a bulk measurement which does not take into account local variations of the elasticity. We suggest that using the elastic modulus of a pure F-actin meshwork in the calculation of *C*_*f*_ gives a better representation of the actual condition of the internal veins. For a cross-linked actin network, values of Young’s modulus were found to be ∼ 5.2 kPa [34], which is approximately three times smaller than what we measured in microplasmodia. Thus, we used *E* = 5.2 kPa for our calculations.

#### Impedance

We can now model a segment of internal flow channel as an electrical circuit, based on Ohm’s law for alternating current (AC), which states that current equals voltage divided by impedance. In the equivalent fluid flow system, this relationship is

$$\begin{array}{c} q=\frac{\Delta p}{\left[Impedance\right]} \end{array}$$ (12)

Impedance (Z) is the opposition that a circuit presents to a current when a voltage is applied. Due to the fact that the shuttle streaming in the slime mold resembles an AC situation, the capacitor exhibits reactance (equivalent to resistance). Thus, its capacitive reactance varies with the applied frequency: Higher frequencies lead to a decrease in reactance.

The dynamics of cytoplasm flow in *P. polycephalum* are driven by pulsatile pressure waves generated by the actomyosin cortex. We therefore need to give the driving pressure gradient Δ*p* the form of a cosine pressure source

$$\begin{array}{c} \Delta p=\Delta p_{0}cos\omega t \end{array}$$ (13)

where Δ*p*_0_ is a constant (the amplitude of input pressure) and *ω* is the (angular) oscillation frequency. We want to obtain the steady state of the circuit. In steady state, the system has completely adjusted to the initial imposed conditions and no further change in flow rate pattern takes place. For further analysis of the fluid equivalent circuit, we used the freeware computer software LTSpice (Linear Technology Corporation), which implements a SPICE simulator of electronic circuits.

The complex impedance of a circuit as shown in Fig 8 is

$$\begin{array}{c} Z=\left(R_{2}+R_{1}\right)\frac{1+j\omega \tau_{1}}{1+j\omega \tau_{2}} \end{array}$$ (14)

with

$$\begin{array}{ccccc} \tau_{1}=\frac{R_{2}R_{1}}{R_{2}+R_{1}}C & & \text{and} & & \tau_{2}=R_{2}C \end{array}$$ (15)

*τ*_1_ and *τ*_2_ are time constants which give an indication of pressure decay. They are related as follows:

$$\begin{array}{c} \tau_{1}=\tau_{2}\frac{R_{1}}{\left(R_{2}+R_{1}\right)} \end{array}$$ (16)

*R*_1_ is the resistance of the vein segment to flow, and *R*_2_ is the loss due to leakage. In contrast to *R*_1_, which can be easily identified from Eq 4, *R*_2_ can not be obtained readily from image sequences. As described above, leakage is defined as *R*_2_ [30], which is due to the complete absence of a vessel wall. *R*_2_ accounts for the amount of cytoplasm lost laterally per unit length and can be interpreted as conductance, i.e., the inverse of an (unknown) resistance. In lymph nodes, which are very permeable vessels, the equivalent resistance is reported to be ∼ 100 times larger than that of non-filtrating lymph ducts [35]. We take a value for *R*_2_ that is 50 times larger than *R*_1_.

#### Cut-off frequency

By definition, the cut-off frequency of an electronic filter is the frequency at which the power output of this circuit has fallen to a given proportion of the power of the admitted frequencies (also termed passband). This is usually at one half of the passband. The corresponding voltage ratio is at $\frac{1}{\sqrt{2}}$, or 3 dB. The transfer function of a 3-element Windkessel circuit is [36]

$$\begin{array}{c} H\left(s\right)=\frac{sR_{2}CR_{1}+R_{1}+R_{2}}{sR_{2}C+1} \end{array}$$ (17)

By setting the magnitude of the transfer function equal to $\frac{1}{\sqrt{2}}$, we obtain the cut-off frequency

$$\begin{array}{c} f_{c}=\frac{\frac{R_{1}+R_{2}}{CR_{1}R_{2}}}{2\pi } \end{array}$$ (18)

For the analysis of one short, unbranching vein segment as given in Fig 8A, we used an average vein radius of *a* = 20 μm, and an average length of *l* = 500 μm, as measured from images. From Eqs 4 and 5, we obtain the fluidic resistance $R_{f}=R_{1}=2.19\times 10^{15}\frac{Ns}{m^{5}}$ and the fluidic capacitance $C_{f}=2.42\times 10^{-16}\frac{m^{5}}{N}$. *R*_2_ was set to 50 times *R*_1_. Inserting these values into Eq 18 gives a cut-off frequency of 0.31 Hz.

The same cut-off frequency can be obtained by modeling the circuit in LTSpice. For use in a circuit simulation program like LTSpice, fluidic values have to be converted into ohm and farad, respectively. The conversion of fluidic units to electrical analogue units can be found in S2 Text. Table 2 gives the parameters for a single tube segment.

**Table 2:** Fluidic and corresponding electric characteristics of single tube segment.

*l* = 500 μm	
*a* = 20 μm	
$C_{f}=2.42\times 10^{-16}\frac{m^{5}}{N}$	*C*_*e*_ = 0.053 F
$R_{1f}=2.19\times 10^{15}\frac{Ns}{m^{5}}$	*R*_1*e*_ = 10 Ω
$R_{1f}=1.09\times 10^{17}\frac{Ns}{m^{5}}$	*R*_2*e*_ = 500 Ω

#### Analytical solution of 3-element Windkessel model

Eq 14 can also be written as

$$\begin{array}{c} Z=R_{1}+\frac{R_{2}}{1+(j\omega R_{2}C)} \end{array}$$ (19)

This complex impedance has a real part (*Z*_*real*_) and an imaginary part (*Z*_*im*_):

$$\begin{array}{c} Z_{real}=R_{1}+\frac{R_{2}}{1+(\omega^{2}{R_{2}}^{2}C^{2})} \end{array}$$ (20)

$$\begin{array}{c} Z_{im}=\frac{{R_{2}}^{2}C\omega }{1+(\omega^{2}{R_{2}}^{2}C^{2})} \end{array}$$ (21)

From these equations, the magnitude of the impedance (|*Z*|), i.e., the ratio of the voltage difference amplitude to the current amplitude, and the phase angle (*θ*) between voltage and current can be obtained.

$$\begin{array}{c} \left|Z\right|=\sqrt{{Z_{real}}^{2}+{Z_{im}}^{2}} \end{array}$$ (22)

$$\begin{array}{c} \theta =\text{arctan}(\frac{Z_{im}}{Z_{real}})\text{in}\;\text{radians} \end{array}$$ (23)

The analytical solution for a 3-element Windkessel as shown in Fig 8, i.e. for a single, non-branching internal vein segment, is as follows. We begin with a driving pressure drop, as was introduced in Eq 13. Its complex form is

$$\begin{array}{c} \Delta p\left(t\right)=\Delta p_{0}e^{i\omega t} \end{array}$$ (24)

The equation for flow rate is, as given by Eq 12,

$$\begin{array}{c} q\left(t\right)=\frac{\Delta p(t)}{Z} \end{array}$$ (25)

or, to use the concept of reactance, we get the complex flow rate

$$\begin{array}{c} q\left(t\right)=\frac{\Delta p_{0}e^{i\omega t}}{Z_{real}+Z_{im}} \end{array}$$ (26)

The imaginary part *Z*_*im*_ of the complex impedance is also termed reactance. The real part of Eq 24 is a cosine function:

$$\begin{array}{c} \Delta p(t)=ℜ\{\Delta p_{0}e^{i\omega t}\} \end{array}$$ (27)

$$\begin{array}{c} =\Delta p_{0}\text{cos}\;\omega t \end{array}$$ (28)

**Fig 10:**
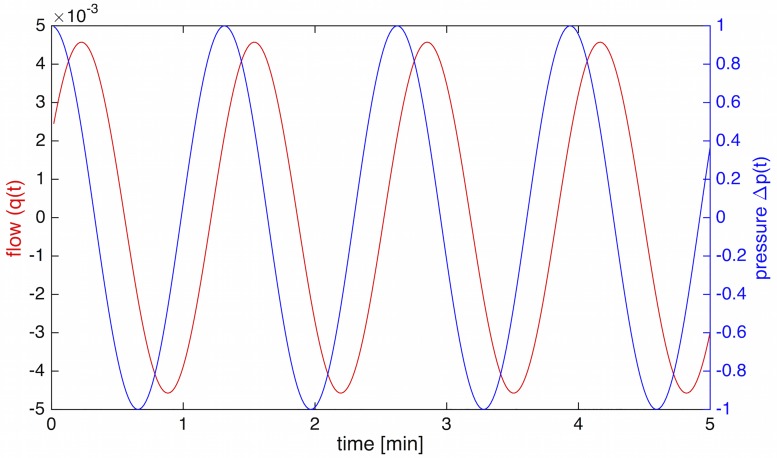
Phase difference between pressure and flow. Pressure (Δ*p*(*t*)) and flow rate (*q*(*t*)). Flow lags *θ* = 62° behind the pressure wave.

Therefore, the flow rate corresponding to this pressure drop is the real part of the complex flow rate of Eq 26:

$$\begin{array}{c} q(t)=ℜ\{\frac{\Delta p_{0}e^{i\omega t}}{Z_{real}+Z_{im}}\} \end{array}$$ (29)

$$\begin{array}{c} =\Delta p_{0}ℜ\{\frac{\left(\text{cos}\;\omega t+i\;\text{sin}\;\omega t\right)\left(Z_{real}-Z_{im}\right)}{{Z_{real}}^{2}+{Z_{im}}^{2}}\} \end{array}$$ (30)

$$\begin{array}{c} =\Delta p_{0}\{\frac{Z_{real}\;\text{cos}\;\omega t+Z_{im}\;\text{sin}\;\omega t}{{Z_{real}}^{2}+{Z_{im}}^{2}}\} \end{array}$$ (31)

which, using trigonometric identities, equals

$$\begin{array}{c} q(t)=\frac{\Delta p_{0}}{\sqrt{{Z_{real}}^{2}+{Z_{im}}^{2}}}\;\text{cos}\;\left(\omega t-\theta \right) \end{array}$$ (32)

$$\begin{array}{c} =\frac{\Delta p_{0}}{\left|Z\right|}\;\text{cos}\;\left(\omega t-\theta \right) \end{array}$$ (33)

with phase angle *θ* as given in Eq 23. *θ* can also be obtained from the LTSpice simulated circuit. In Fig 10, the relationship between pressure (Eq 28) and flow (Eq 33) is shown.

To further elucidate the nature of impedance and phase angle, we can discuss the limiting behavior of these equations. If *C* = 0, *Z*_*im*_ becomes 0 and *Z*_*real*_ becomes *R*_1_+ *R*_2_. This means that *θ* = 0, i.e. there is no phase difference between flow and pressure. Eq 32 would then become

$$\begin{array}{c} q\left(t\right)=\frac{\Delta p_{0}}{\sqrt{{\left(R_{1}+R_{2}\right)}^{2}}}\;\text{cos}\;\left(\omega t\right) \end{array}$$ (34)

indicating a purely resistive circuit. If *R*_1_ = 0, *Z*_*im*_ remains unaffected and $Z_{real}=\frac{R_{2}}{1+(\omega^{2}R_{2}^{2}C^{2})}$. Thus, the flow would not differ much. However, if *R*_2_ = 0, *Z*_*im*_ = 0 and *Z*_*real*_ = *R*_1_. This results in *θ* = 0, and $q\left(t\right)=\frac{\Delta p_{0}}{\sqrt{R_{1}^{2}}}\;\text{cos}\;\left(\omega t\right)$, i.e. a circuit that is also purely defined by resistance.

#### Dimensionless parameters

It is also possible to characterize the relationship between pressure and flow wave in the 3 element Windkessel model using the two dimensionless parameters $\tilde{r}=\frac{R_{1}}{R_{2}}$ and $\tilde{c}=CR_{2}\omega$, and the term $\frac{1}{R_{2}}$ [37]. Expressed in these terms of interest, the flow then becomes

$$\begin{array}{c} q_{rel}=(\frac{(\frac{1}{R_{2}}(\tilde{r}+1)+\frac{1}{R_{2}}\tilde{r}{\tilde{c}}^{2})\Delta p_{0}}{{\left(\tilde{r}+1\right)}^{2}+{\tilde{r}}^{2}{\tilde{c}}^{2}})\text{cos}\;\left(\omega t-\theta_{rel}\right) \end{array}$$ (35)

The parameter $\frac{1}{R_{2}}$ acts as a scaling factor for the flow. To simplify things further, we introduce the dimensionless flow $\tilde{q}$.

$$\begin{array}{c} \tilde{q}=\frac{qR_{2}}{\Delta p_{0}}=\frac{\tilde{r}+1+\tilde{r}{\tilde{c}}^{2}}{{\left(\tilde{r}+1\right)}^{2}+{\tilde{r}}^{2}{\tilde{c}}^{2}}\;\text{cos}\;\left(\omega t-\theta_{rel}\right) \end{array}$$ (36)

The relative phase shift *θ*_*rel*_ between pressure and flow can also be expressed in terms of dimensionless parameters and then reads as follows:

$$\begin{array}{c} \theta_{rel}=\text{arctan}\;(\frac{\tilde{c}}{\left(\tilde{r}+1\right)+\tilde{r}{\tilde{c}}^{2}}) \end{array}$$ (37)

**Fig 11:**
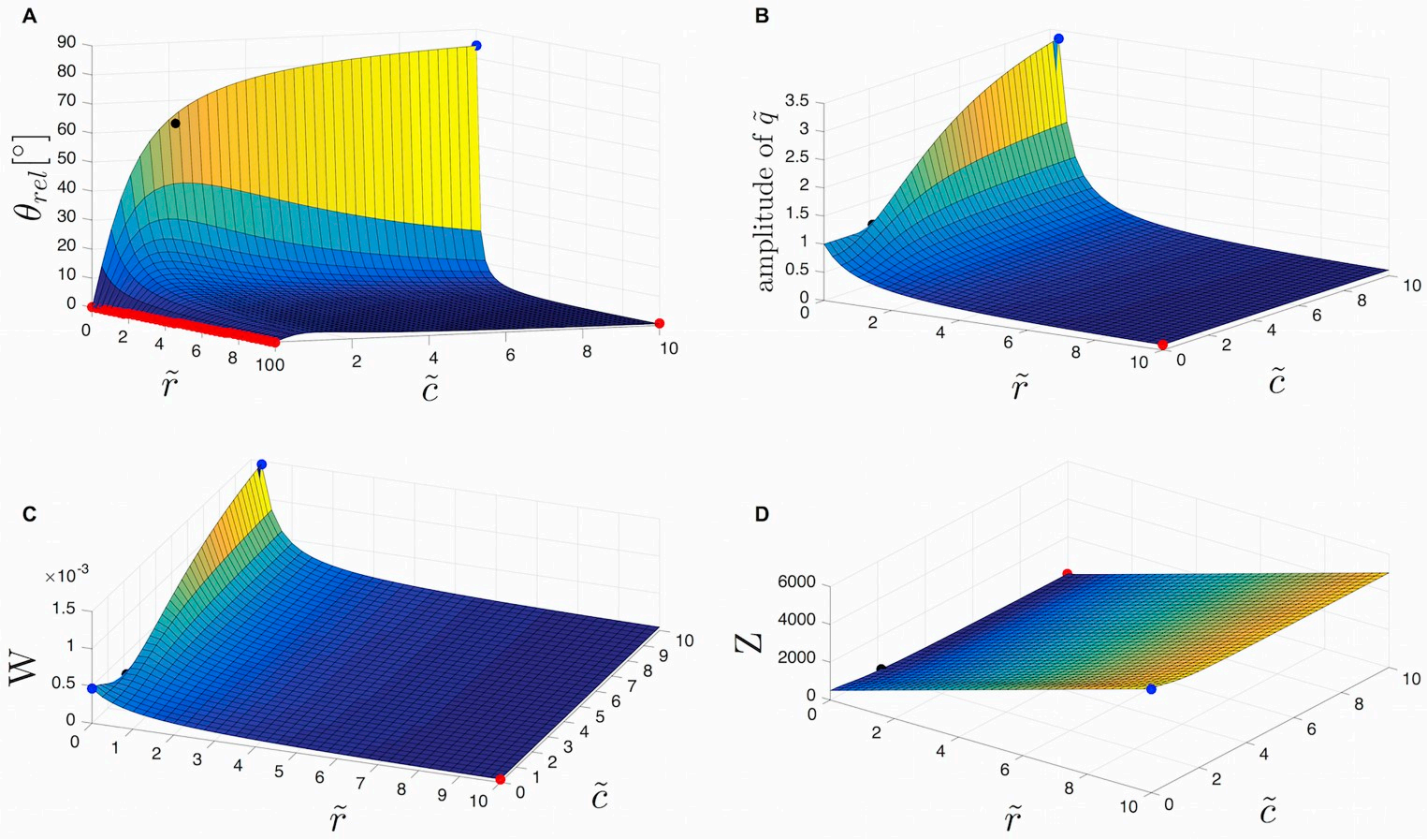
Dependence of phase angle, flow amplitude, power dissipation and impedance on the dimensionless parameters $\tilde{r}$ and $\tilde{c}$. Illustration of how *θ*_*rel*_ (A), amplitude of the flow $\tilde{q}$ (B), power dissipation *W* (C), and impedance *Z* (D) vary with the dimensionless parameters $\tilde{r}$ and $\tilde{c}$. Red dots = local minima, blue dots = local maxima, black dots = corresponding values for calculated $\tilde{r}$ and $\tilde{c}$.

Another factor that can be assessed is power dissipation (*W*), which has a steady and an oscillatory part [38]. The steady part is the product of mean pressure and mean flow and takes on very small values, hence it is disregarded in the following. The oscillatory power dissipation over one oscillation period is given as

$$\begin{array}{c} W=\frac{1}{2}Q^{2}\left|Z\right|\;\text{cos}\;\theta \end{array}$$ (38)

where *Q* is the amplitude of *q*_*rel*_ (Eq 35). Fig 11 shows how phase angle *θ*_*rel*_, flow amplitude, power dissipation and impedance vary with $\tilde{r}$ and $\tilde{c}$.

*θ*_*rel*_ has a maximum at 84.3°, when $\tilde{r}$ is zero and $\tilde{c}$ is maximal. The effect of an increasing ratio of $\tilde{r}$ is the reduction of the phase angle between pressure and flow (Fig 11A). However, the effect is most pronounced for small $\tilde{r}$. For one vein segment (and one characteristic oscillation frequency), $\tilde{r}$ is 0.02 and $\tilde{c}$ is ∼ 2. An increasing ratio of $\frac{R_{1}}{R_{2}}$ leads to a reduced amplitude of the flow wave (Fig 11B). As $\tilde{r}$ becomes small and $\tilde{c}$ becomes large, there is a peak in the amplitude of the flow. Thus, we can state that the flow rate increases with the vessel compliance. A higher elasticity, in combination with low-frequency pulsatile flow, leads to an enhanced flow rate. This has been established [23, 39].

In a mesoplasmodium, the uroid generates sinusoidal pressure waves with different oscillation frequencies, but we observe net forward flow of cytoplasm (see Fig 3). Thus, when simulating equivalent electric circuits, we model this by superimposing a DC signal over the AC signal. This grants net forward flow without necessitating diodes (electrical) or valves (biological).

The forward migration of mesoplasmodia is based on myosin II-driven rhythmic back-and-forth oscillations of the actin cytoskeleton. These contractions drive the observed cytoplasm flow along the longitudinal axis of the amoeba. The entire locomotion process is far from completely understood, but an asymmetry of the cell in terms of a softness gradient [9] seems to play a big role, as well as substrate adhesion [4, 40] and the transition from endo- to ectoplasm and vice versa.

Kirchhoff’s current law (KCL) states that charge cannot accumulate at the nodes of a circuit. In fluid dynamics, that means that the amount of fluid which flows into one end of a pipe equals the outflow. Continuing with our analogy that the elastic tube walls act like capacitors, we have to take into consideration that capacitance *C* affects the total volume of the tube. Therefore, the flow rate at the entrance of the tube may not be the same as that at the exit, because some of the flow may inflate the tube upon entering it, thus reducing the output; and some of the exit flow may result from a part of the vessel deflating. We therefore have to take retrograde flow of endoplasm into account. This means that forward flow through the internal veins enters the frontal region, then bounces off the membrane and transforms into backwards flow.

**Fig 12:**
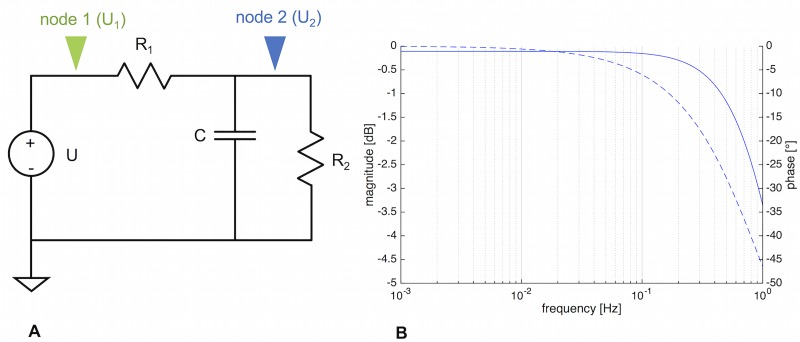
LTSpice schematic and resulting Bode plot of a three-element Windkessel. A) Schematic of 3-element Windkessel. U = voltage source, *R*_1_, *R*_2_ = resistors, *C* = capacitor. Green arrow = node 1, blue arrow = node 2. B) Bode plot of the circuit, taken at node 2. The cut-off frequency can be obtained mathematically, or it can be found from the Bode plot at the frequency where the gain falls below—3 dB.

The LTSpice schematic of the single tube (a single 3-element Windkessel) is given in Fig 12A.

An AC analysis results in a Bode plot (see Fig 12B), and at the -3 dB mark, the cut-off frequency *f*_*c*_ can be read off. It can also be determined via Eq 18. Both methods give *f*_*c*_ = 0.31 Hz (corresponding to an oscillation with T = 3.26 s). Therefore, a single tube segment does act as a low-pass filter, but only at frequencies way above what we observe in slime mold mesoplasmodia.

A slight lag between the voltage at node 1 and node 2 (see Fig 12A) can be observed. Node 1 represents the input voltage (*U*_1_), whereas at node 2, the voltage as influenced by the circuit’s components can be extracted (*U*_2_). The phase angle between both voltage signals is $\phi_{U_{1}U_{2}}=2.4{}^{\circ}$. This means that along the vein segment (we assumed a length of 500 μm), the voltage (pressure) signal is slightly delayed. This delay of the pressure wave along the tube is a feature of a distributed model, in which spatial dimension does play a role. Furthermore, when flow is occurring in an elastic tube, the local change of pressure propagates like a wave crest down the tube. In a rigid tube, a local change of pressure would occur instantaneously everywhere within the tube.

In summary, we find that a single tube section of internal vein can not account for the observed frequency selection, although it acts as a low-pass filter. Pressure precedes the flow by 62°, and along the length of the segment, the pressure wave is slightly delayed between the entrance and the exit of the tube.

#### Analysis of four coupled segments

**Fig 13:**
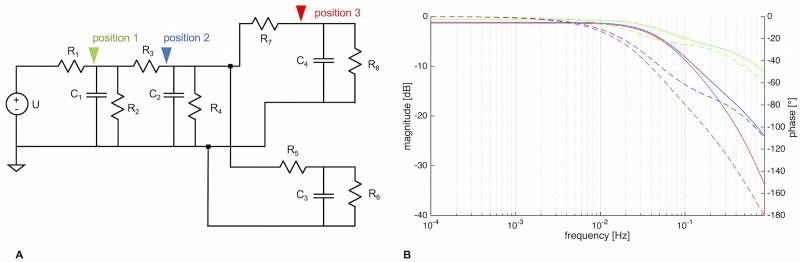
LTSpice schematic and resulting Bode plot of four three-element Windkessel. A) LTSpice schematic of four cascaded 3WK elements. U = voltage source, R = resistor, C = capacitor. B) Bode plots obtained at positions 1 (green), 2 (blue), and 3 (red). The more segments are added, the steeper does the filter cutoff become. Solid lines = magnitude [dB], dashed lines = phase [°].

Since one single 3-element Windkessel (3WK) model was not sufficient to describe the dynamics within the mesoplasmodium, we constructed a branching vein model as shown in Fig 9C. The corresponding LTSpice circuit is given in Fig 13. The model circuit consists of four 3WK elements, branching in a Y-shaped fashion. The respective values were the same as in the single 3WK model (see Table 2).

The arrangement of Fig 13 is a filter cascade, connecting multiple low-pass filters. An AC analysis reveals different cutoff-frequencies throughout the circuit, as given by the three different traces (green, blue, red) in Fig 13. *f*_*c*_ of position 3 (red), the end point of the branching structure, was found to be 0.033 Hz, which is in accordance with our data (see Fig 7). This frequency is ten times lower than that of one single 3WK element. *f*_*c*_ of position 1 (green) was 0.058 Hz, and *f*_*c*_ of position 2 (blue) was 0.036 Hz. Thus, the further the distance from the uroid (the input signal) and the further along the filter cascade, the better does the Y arrangement reflect the real situation within the slime mold. In other words, the length of the internal vein plays a major role in the low-pass filtering process. This explains why very small mesoplasmodia do not exhibit frequency selection.

**Fig 14:**
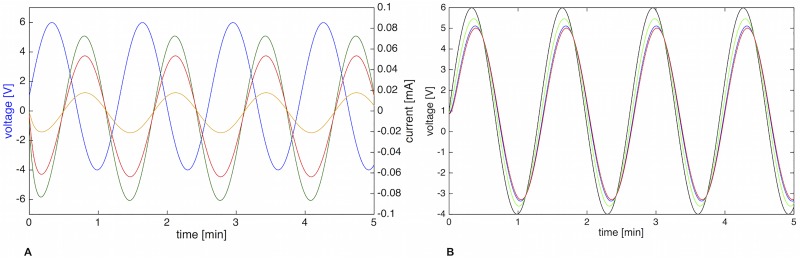
Input voltage and currents at three different positions; voltage at different positions. A) Input voltage (blue curve) and current through *R*_1_ (green curve), *R*_3_ (red curve), and *R*_7_ (orange curve), respectively. B) Voltage measured at positions 1 (green), 2 (blue), and 3 (red). Input voltage = black curve.

Within the branched four 3WK model, flow and pressure wave do not occur simultaneously. Rather, pressure (voltage) leads the flow (current), see Fig 14. The phase difference between input pressure and flow at position 3 is *θ* ≈ 127°.

**Fig 15:**
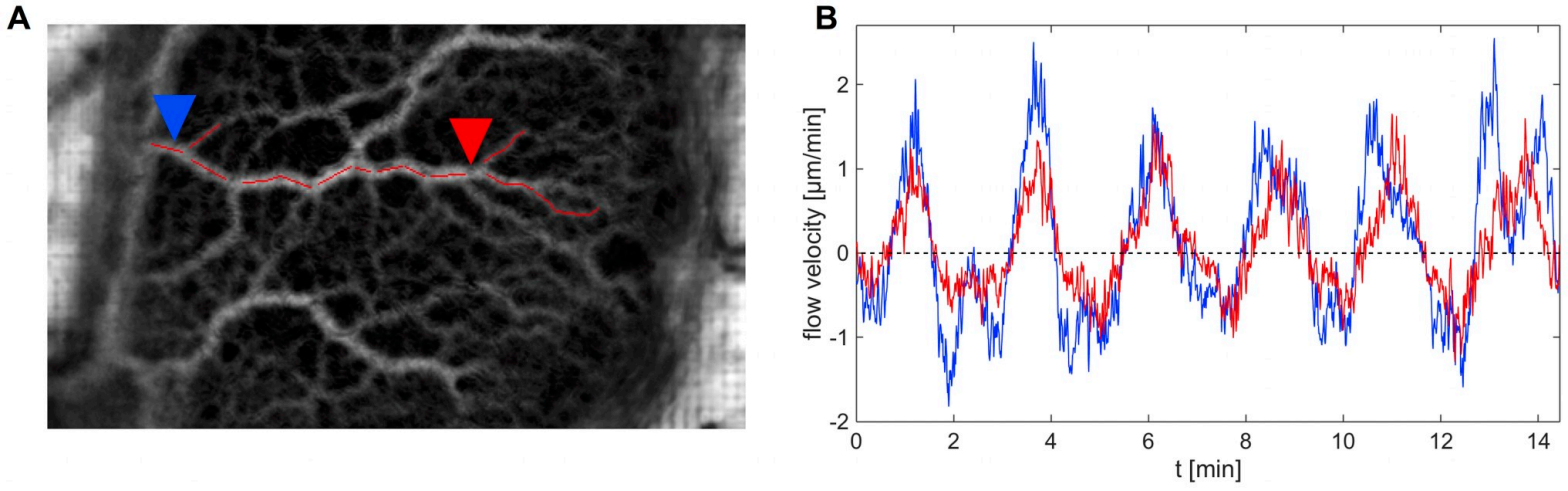
Flow velocity at different positions along a vein. A) Time series (standard deviation) of a mesoplasmodium with a major vein highlighted in red. Blue and red arrowheads denote positions at which internal flow was measured. B) Flow velocity at the two indictated positons in A along a forward moving mesoplasmodium.

Along the Y-shaped cascade, the voltage (i.e. pressure wave) is slightly delayed. This can be seen in Fig 14B. We found a phase angle of *ϕ* ≈ 18° between the input voltage signal and voltage at position 3 (red arrow in Fig 13). Fig 15 shows flow velocity measurements at two different positions along an internal vein. It becomes obvious that the flow waves lag between positions, more specifically, that the flow occurs first closer to the uroid and then travels towards the front.

### Modeling chemotaxis

**Fig 16:**
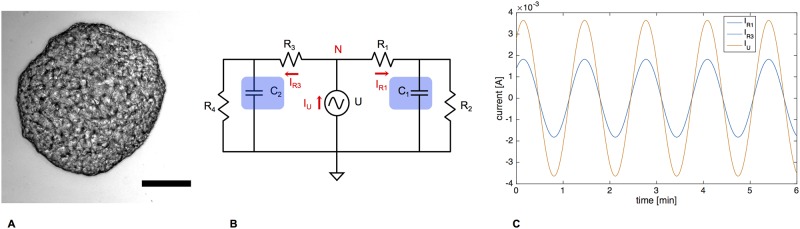
Case 1: Stationary, unpolarized microplasmodium. A) Stationary, unpolarized microplasmodium. Scale bar = 100 μm. B) Symmetrical model circuit for an oscillating but stationary microplasmodium. C = capacitors, R = resistors, U = voltage source, I = current, N = node. C) Current flowing through the right side as taken at *R*_1_ and *R*_3_ (blue) and the voltage source (*I*_*U*_, orange).

Our Y-shaped filter cascade works very well to describe the dynamics within a moving mesoplasmodium. However, our lumped model can also be adapted and used to explain how a slime mold starts to migrate in one particular direction, or how it could change its direction. This again introduces a spatial (or distributed) component to the otherwise lumped model. To model asymmetry introduced by polarization and (chemotactic) migration, we arranged two 3WK symmetrically side by side, with a voltage source as oscillating driving pressure (without DC offset) in the middle (see Fig 16). This circuit is symmetrical, meaning the same impedance is present on both sides. The current which flows through the entire right side of the circuit can be obtained by measuring it at *R*_1_, and for the entire left side, at *R*_3_, respectively.

#### Case 1: Stationary microplasmodium

In the first case, we want to consider a stationary, non-migrating microplasmodium. It is not motionless, because it exhibits rhythmic oscillations [14]. However, the cell is unpolarized. The actin cortex which surrounds the whole microplasmodium and which lies directly underneath the plasma membrane is homogenous [8]. This condition is represented by the circuit as shown in Fig 16, with the resistors and capacitors on both sides set to the same values, respectively. The AC voltage source represents the rhythmic contraction-relaxation pattern which is the basis of the pressure waves. At node N, according to Kirchhoff’s current law (KCL), the sum of currents flowing into the node equals the sum of currents flowing out of the node, i.e. *I*_*U*_ = *I*_*R*1_ + *I*_*R*2_. This circuit arrangement can best be compared to a valve that connects two identical balloons. Because the balloons are the same, the inflow (*I*_*U*_) splits up evenly into two flows to the right (*I*_*R*1_) and the left (*I*_*R*3_). Fig 16C shows *I*_*R*1_ and *I*_*R*3_, which have the exact same phase and amplitude because of the circuit’s symmetry. Since both currents are flowing out of the node, their direction is also identical. Averaged over 5 oscillation periods, *I*_*U*_ gives an average current of almost zero (9.85 ⋅ 10^−6^ A). In the slime mold, the total volume of cytoplasm remains constant, and thus KCL also applies to the fluidic system. Because there is no difference in elasticity, each side receives the same amount of cytoplasm. This is equivalent to a stationary, oscillating plasmodium.

#### Case 2: Cell polarity: Introducing asymmetry

**Fig 17:**
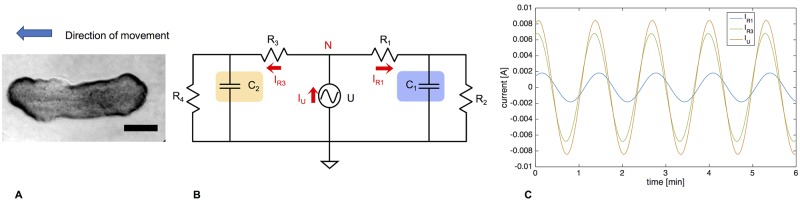
Case 2: Polarized, migrating plasmodium. A) Migrating, polarized microplasmodium. Scale bar = 50 μm. B) Symmetrical model circuit for a motile microplasmodium. C = capacitors, R = resistors, U = voltage source, I = current. C) Current flowing through the right side as taken at *R*_1_ (blue) and the left side as taken at *R*_3_ (green), and current flowing through the voltage source (*I*_*U*_, orange).

In the second case, we discuss polarization and subsequent start of migration. We argue that the onset of locomotion is due to the development of an elasticity gradient throughout the cell. It is known that the front of a migrating plasmodium is softer than the back. Local softening of the actin cortex plays a big role in the amoeboid movement of *P. polycephalum* [3, 9]. The ultrastructural analysis of a migrating mesoplasmodium also shows a denser, more organized actin cortex in the back and a weaker cortex in the front [8]. A strong actin cortex in the trailing edge and a soft actin cortex at the leading edge are also a requirement for locomotion in *D. discoideum* [10]. The ubiquitous oscillations are always present (as represented by the AC voltage source in Fig 17), but the mesoplasmodium is now polarized and subsequently moves.

The components of importance in the equivalent circuit are the capacitors. *C*_2_ was set to five times *C*_1_, which corresponds to a higher elasticity at the front. Due to the higher capacitance, the reactance of capacitor *C*_2_ is reduced and the current increases in the left side of the circuit. In an electric capacitor, the larger the capacitance, the more charge has to flow to build up a particular voltage, and the higher the resulting current will be. Returning to the aforementioned example of a valve connecting two balloons, we now have the situation of the left balloon being five times softer than the right balloon. Thus, due to the smaller resistance, the inflow (*I*_*U*_) splits up into a low flow to the right (*I*_*R*1_) and an enhanced flow to the left (*I*_*R*3_). In terms of fluidics, this means a higher flow rate towards the left side of the circuit. Averaged over 5 oscillation periods, *I*_*U*_ gives an average current of 3.09 ⋅ 10^−4^ A, which is ∼ 30 times more than in the stationary case. Increasing the capacitance of the left capacitor (*C*_2_) to five times that of *C*_1_ (right side) also results in a current through the left side (*I*_*R*3_) which is fifty times higher than *I*_*R*1_.

**Fig 18:**
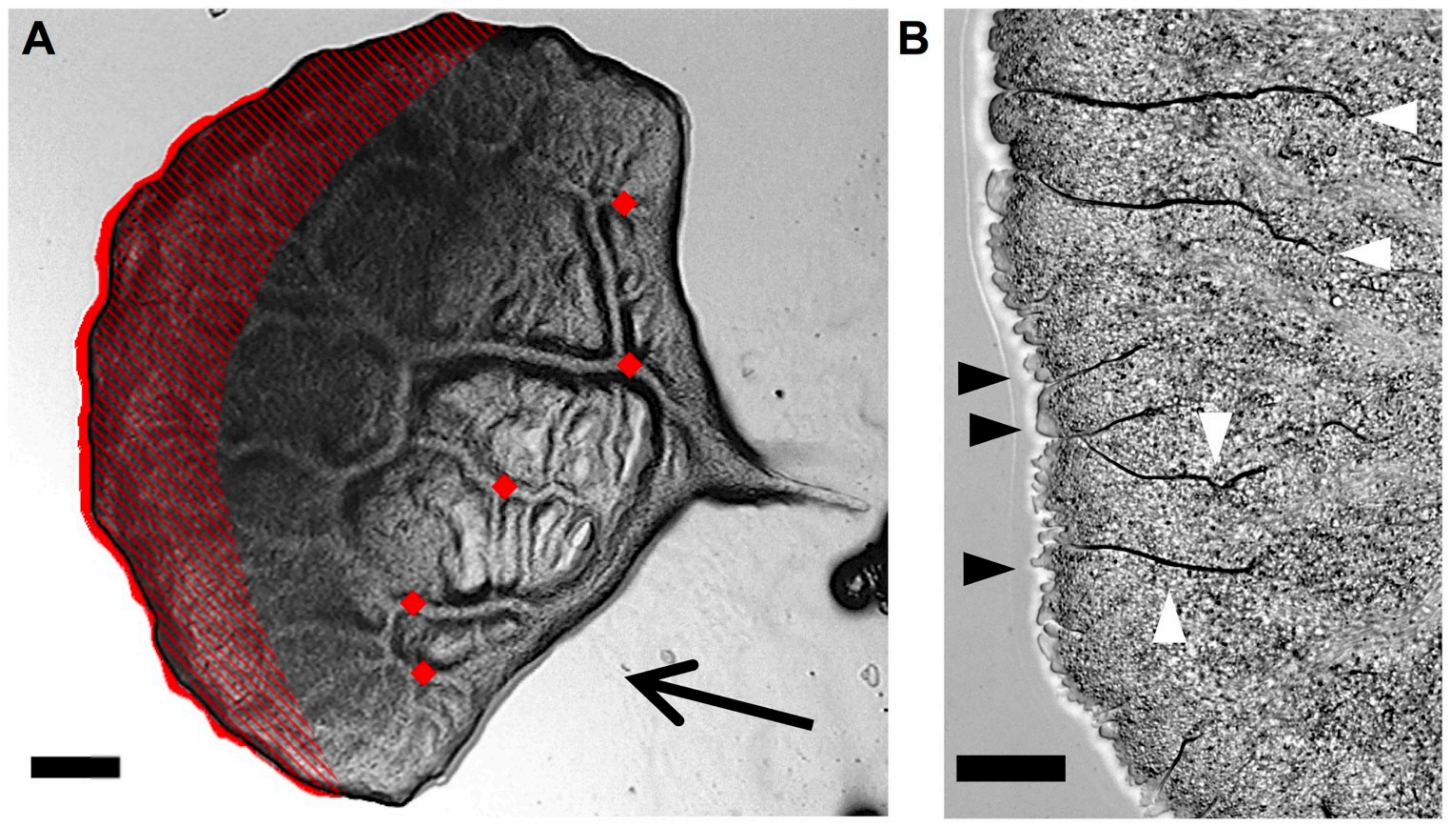
Volumetric flow rates and micromorphology in a migrating mesoplasmodium. A) Migrating mesoplasmodium. Scale bar = 200 μm. Overlay of two images taken at an interval of ∼ 3 min. From the solid red area, we calculated the overall volumetric flow rate (which corresponds to *I*_*U*_). Red diamonds designate points on main internal veins at which flow rates were measured and which correspond to *I*_*R*1_. Hatched area indicates frontal sheet. B) Higher resolution of the front of a migrating mesoplasmodium. Scale bar = 50 μm. White arrows point to membrane folds, black arrows to blebs.

To verify this outcome, we calculated the volumetric flow rates in a migrating mesoplasmodium (see Fig 18A). As mentioned before, both the volume and the shape of a mesoplasmodium remain constant over the time scale of observation. Also, mesoplasmodia travel on straight trajectories. Thus, by overlaying the image at the start of a time sequence with the last image in the stack, we obtain the solid red area in Fig 18A. This is the area which is accumulated during the time interval of interest. Assuming a constant height of 100 μm, we obtain a volumetric flow rate of *Q*_*total*_ = 9.8 ⋅ 10^5^ μm^3^/min.

*Q*_*total*_ corresponds to *I*_*U*_. Next, we measured the flow rate in the back of the mesoplasmodium, which is the equivalent to *I*_*R*1_, i.e., the flow through the right side of the circuit. The red diamonds in Fig 18A show the points at which flow was measured. We calculated the volumetric flow rate as follows (Eq 39) to obtain the flow for each vein from the flow velocity *v*, which was obtained from optical flow analysis, and the cross-sectional areas *A* of the veins, assuming a radius of *a* = 20μm.

$$\begin{array}{c} Q=vA \end{array}$$ (39)

Most of the flow in the uroidal region passes through those veins. The sum of the flows amounts to *Q*_*back*_ = 2.8⋅10^4^ μm^3^/min. By applying KCL, we find the flow rate through the frontal area as *Q*_*front*_ = *Q*_*total*_ − *Q*_*back*_ = 9.5 ⋅ 10^5^ μm^3^/min. *Q*_*front*_ corresponds to *I*_*R*3_, the flow through the left side of the circuit. *Q*_*front*_ is ∼ 30 times larger than *Q*_*back*_, which is in good agreement with the electric circuit model (where *I*_*R*3_ = 30 × *I*_*R*1_).

At this point, the interdependence between ultrastructure and flow needs to be briefly discussed. For a more detailed investigation, see [8]. As can be seen in Fig 18B, the frontal area of the mesoplasmodium (hatched area in Fig 18A) possesses many deep folds and creases which most likely represent a membrane reservoir. Like inflating a wrinkled balloon, when cytoplasm flows into this area, the membrane easily yields. Furthermore, blebbing can be observed along the whole front. Other effects at play in the frontal sheet are height fluctuations and gel-sol transitions. All of these processes have their own dynamics and will affect the flow.

In summary, we propose a mechanism which, induced by a chemotactic signal transduction (or internal cues), leads to a local softening of the actin cortex at the site of the chemoattractant stimulus. The capacitance of the vessels increases, which, in turn, enhances the flow in the affected vessels. A correlation between more elastic vessels and enhanced volumetric flow has also been shown to occur in blood vessels [41]. This leads to an increased mass transport towards the left side (see Fig 17) and less flow towards the right, so that the slime mold would effectively move to the right.

## Conclusion and outlook

Recently, *P. polycephalum* has become the focus of research on the fundamental mechanisms of cognition and decision-making (for a review, see [42]). The question of how the slime mold processes information in the absence of a nervous system is still unanswered. For its apparent reminiscential capabilities, a model based on memristors has been put forward [43]. The selection of frequencies is an example of hydrodynamic information processing. We speculate that cytoplasm flow is a means for the slime mold to transmit information throughout its cell body, and to process this information so that an observable change in behavior takes place. The input signal, e.g., a chemotactic stimulus, could cause a change in the oscillation pattern which is then transmitted through the entire organism. On the other hand, based on internal cues (e.g. the cell cycle or nutritional status), the slime mold could control, globally or locally, the radius and stiffness of its veins via the actin cytoskeleton. This would change the fluidic properties (resistance, capacitance), and thus the flow. The internal flow pattern, in turn, changes the morphology and behavior of the organism.

To our knowledge, no previous modeling has been done using the lumped parameter approach presented here. As of late, several models regarding the mechanical properties of *P. polycephalum* and their implications for pattern formation and oscillations have been proposed. By integrating experimental data into a quantitative framework, these models can shed light on the spatio-temporal coordination of cell motility. Some variables are hard to measure experimentally, which is a strong motivation for the use of physical models, including our lumped model. Under this premise, various models have been constructed. Viscoelastic or poroelastic models were able to reproduce many experimentally observed movement patterns, for example [44–46]. Zhang and coworkers [47] have also measured endoplasm velocity, but have extended their measurements to include traction stress and calcium. They found that asymmetric spatio-temporal patterns of endoplasmic flow lead to a higher migration speed than purely symmetrical patterns, a result that is in accordance to our findings. However, most of those models rely on accurate biological data, e.g. biochemical processes and calcium dynamics, which are still not very well understood in *P. polycephalum*. An advantage of our model is that it does not require a great deal of biological detail, e.g. the dynamics and quantitative rates of chemical reactions. As we learn more about the inner workings of the slime mold, such detail can be used to amend and expand our model.

We present an efficient and fast way in which signals can be processed without the necessity for complicated biochemical signal transduction pathways. Our model explains how *P. polycephalum*’s complex behavior is based on intracellular cytoplasm flow. Furthermore, local softening of the actin cortex can account for an increased flow towards that area and a subsequent introduction of asymmetry, so that locomotion can be initiated.

## Supporting information

S1 Text Optical flow analysis. PDF containing a detailed description of the optical flow analysis after Horn and Schunck.
(PDF)

S2 Text Conversion of fluidic to electric units. PDF containing the equations used to convert fluidic units to equivalent electrical units for use with LTSpice.
(PDF)

S1 Table Locomotion parameters for six mesoplasmodia. Mean locomotion speed as gathered from kymographs, uroid half angle, area covered by mesoplasmodium, circularity, period of area oscillations, and ratio of membrane extension to retraction time.
(PDF)

S1 Fig Trajectories of four mesoplasmodia. Trajectories of the center of mass of four migrating mesoplasmodia. A straight path is maintained for hours. Inset: Magnification of a detail of a trajectory. The centre of mass of a mesoplasmodium moves along a cycloid path.
(TIFF)

S2 Fig Circularity over time. Circularity *f*_*circ*_ over time for six mesoplasmodia.
(TIFF)

S3 Fig Circularity and frontal membrane velocity over time. Phases of high circularity correspond to a slowing of locomotion.
(TIFF)

S1 Movie Video of a mesoplasmodium showing frequency selection. (MOV)
