# Supplementary material for: A lumped parameter model of endoplasm flow in Physarum polycephalum explains migration and polarization-induced asymmetry during the onset of locomotion
Source: PLoS One. 2019 Apr 23;14(4):e0215622. doi: 10.1371/journal.pone.0215622 (PMC6478327; doi:10.1371/journal.pone.0215622)
Supplement: S1 Text — PDF containing a detailed description of the optical flow analysis after Horn and Schunck. (PDF) [file pone.0215622.s001.pdf]

A lumped parameter model of endoplasm flow in *Physarum polycephalum* explains migration and polarization-induced asymmetry during the onset of locomotion

- Supporting Information -

Christina Oettmeier[1*], Hans-Günther Döbereiner[1]

1 Institute for Biophysics, University of Bremen, Otto-Hahn-Allee 1, 28359 Bremen, Germany

* coettmeier@biophysik.uni-bremen.de

SI Materials and Methods

Optical flow analysis

The calculation of the optical flow, the apparent movement of particles in image sequences, is used to analyze where and when endoplasm is flowing in order to gain a better understanding of the internal veins and their role in the locomotion of *P. polycephalum* mesoplasmodia. We utilized particles which are naturally flowing within the internal veins of the slime mold to calculate flow speed. The advantage of this method is that no foreign particles have to be inserted. Prior to the optical flow analysis, image sequences were smoothed by a Gaussian filter in order to remove noise. Optical flow is based on calculating the motion of particles between two frames.The result is a vector field. A brightness constraint is implied, assuming that a particle at position (x, y, t) with an intensity of $I(x, y, t)$ moves by Δx and Δy in sequential images (Δt), but that the overall brightness remains constant (equation 1)

$$I(x, y, t) = I(x + \Delta x, y + \Delta y, t + \Delta t) \tag{1}$$

which is the same as

$$I_x \cdot u + I_y \cdot v + I_t = 0 \tag{2}$$

Using partial derivatives and assuming small particle movements from one frame to the next, equation (1) can be developed to (equation 3)

$$\frac{\partial I}{\partial x} V_x + \frac{\partial I}{\partial y} V_y + \frac{\partial I}{\partial t} = 0 \tag{3}$$

This, however, is not enough to calculate the velocity components u and v since they are both unknowns, making it an ill-posed problem. Additional constraints are necessary. We used the method first described by Horn and Schunck (1981) [1], whereby a smoothness constraint is introduced to obtain the optical flow components. It is assumed that the change in the vector field is supposed to be minimal and that the objects move coherently through the frames. Taking into account both equation

(1) and the smoothness constraint (second term in equation 4), an energy functional (equation 4) can be proposed which then has to be minimized.

$$E(u,v) = \iint [(I_x u + I_y v + I_t)^2 + \alpha(|\nabla u|^2 + |\nabla v|^2)]dxdy \tag{4}$$

α, a global regularization constant, has to be chosen according to the data quality, i.e. image intensities, type of motion and the amount of noise affecting the image sequence [2]. For our experiments, a value of $\alpha = 1$ was chosen. Equation 4 is now solved for the vectors u and v using the Jacobi method [3], and the vectors are scaled in space and time accordingly. The analysis of the optical flow was performed using the software MATLAB2015b (The Mathworks) with the Image Processing Toolbox. Obtaining a vector field with scaled flow velocities v_{flow} was done using equation 5.

$$v_{flow} = \sqrt{u^2 + v^2} \cdot sgn(u) \tag{5}$$

This is permissible because forward moving mesoplasmodia do not deviate from a straight path, and major veins run mostly parallel to the direction of movement. The cytoplasm flow responsible for locomotion is back and forth, i.e. parallel to the axis of motion. Therefore, flow in the direction perpendicular to the direction of movement can be neglected. The resulting vector field was stored as an image, with every pixel containing v_{flow} at that pixel's location. The standard deviation of all images in a stack was calculated (Z-projection) in FIJI. Each of the output image's pixels contains the standard deviation over all images in the stack at the particular pixel location. Areas with strongly fluctuating flow thus become visible (high standard deviation), whereas areas with little flow variation have a low standard deviation and appear dark. From a Z-projection, the location of a vein was obtained as a segmented line. Along this vein, a kymograph was performed. The kymograph contains the flow velocities along the vein.

References

[1] Horn BKP, Schunck BG. Determining optical flow. Artif Intell. 1981 17(1-3):185–203.

[2] Ng L, Solo V. A data-driven method for choosing smoothing parameters in optical flow problems. IEEE ICIP97.1997;3:3060–363. doi: 10.1109/ICIP.1997.632117

[3] Bronštejn IN, Semendjaev KA. Handbook of mathematics. Springer 2013
