# Supplementary material for: A lumped parameter model of endoplasm flow in Physarum polycephalum explains migration and polarization-induced asymmetry during the onset of locomotion
Source: PLoS One. 2019 Apr 23;14(4):e0215622. doi: 10.1371/journal.pone.0215622 (PMC6478327; doi:10.1371/journal.pone.0215622)
Supplement: S2 Text — PDF containing the equations used to convert fluidic units to equivalent electrical units for use with LTSpice. (PDF) [file pone.0215622.s002.pdf]

A lumped parameter model of endoplasm flow in *Physarum polycephalum* explains migration and polarization-induced asymmetry during the onset of locomotion

# - Supporting Information -

Christina Oettmeier[1*], Hans-Günther Döbereiner[1]

1 Institute for Biophysics, University of Bremen, Otto-Hahn-Allee 1, 28359 Bremen, Germany

* coettmeier@biophysik.uni-bremen.de

SI Materials and Methods

Electronic – hydraulic analogy

For use in a circuit simulation program like LTSpice, fluidic values have to be converted into ohm and farad, respectively. The conversion of fluidic units to electrical analogue units was done as follows: To obtain meaningful electronic capacitances and resistances (C_e and R_e, respectively), we introduce the following equations and definitions.

$$R_f = R_0 R_e \tag{1}$$
$$C_f = C_0 C_e \tag{2}$$
$$t_f = t_0 t_e \tag{3}$$
$$t_0 \overset{!}{=} 1 \tag{4}$$
$$R_{1e} \overset{!}{=} 10\ \Omega \tag{5}$$
$$R_{2e} \overset{!}{=} 800\ \Omega \tag{6}$$

We arbitrarily set the electric resistance R_{1e} to 10 Ω, and $R_{2e} = 800\ \Omega$ and keep the time scales identical. We now have to find the equivalent electric capacitance C_e. Both the fluidic and the electric system are equivalent and coupled via time ($t_e = t_f$), thus we can write

$$R_f C_f = R_e C_e \tag{7}$$
$$(R_0 R_e)(C_0 C_e) = R_e C_e \tag{8}$$
$$C_0 = \frac{R_e}{R_f} \tag{9}$$
$$R_f = R_{1f} + R_{2f} \tag{10}$$
$$R_e = R_{1e} + R_{2e} \tag{11}$$

C_0 can then be reintroduced into equation (2), and the equation can be solved for C_e:

$$C_e = \frac{C_f}{C_0} \tag{12}$$

$$= \frac{C_f(R_{1f} + R_{2f})}{(R_{1e} + R_{2e})} \tag{13}$$

$$= 0.053 \text{ F} \tag{14}$$

The capacitance thus becomes 0.053 F. These values were calculated for one single tube segment. As mentioned above, the time constant τ remains the same, in the electrical as well as in the fluidic system.
