# Supplementary material for: A lumped parameter model of endoplasm flow in Physarum polycephalum explains migration and polarization-induced asymmetry during the onset of locomotion
Source: PLoS One. 2019 Apr 23;14(4):e0215622. doi: 10.1371/journal.pone.0215622 (PMC6478327; doi:10.1371/journal.pone.0215622)
Supplement: S1 Table — Mean locomotion speed as gathered from kymographs, uroid half angle, area covered by mesoplasmodium, circularity, period of area oscillations, and ratio of membrane extension to retraction time. (PDF) [file pone.0215622.s003.pdf]

A lumped parameter model of endoplasm flow in *Physarum polycephalum* explains migration and polarization-induced asymmetry during the onset of locomotion

# - Supporting Information -

Christina Oettmeier[1*], Hans-Gnther Dbereiner[1]

1 Institute for Biophysics, University of Bremen, Otto-Hahn-Allee 1, 28359 Bremen, Germany

* coettmeier@biophysik.uni-bremen.de

SI Table 1

Locomotion parameters for six mesoplasmodia.

Table 1: **Locomotion parameter of several satellites.** $\bar{v}_{kymo}$ **= Mean locomotion speed as gathered from kymographs.** θ **= uroid half angle.** $area$ **= area covered by satellite.** f_{circ}**= circularity.** T_{area} **= period of area oscillations.** $\frac{t_{ext}}{t_{ret}}$ **= ratio of membrane extension to retraction time.**

$\bar{v}_{kymo}$ [µm/min]	θ [°]	$area$ [mm^2]	f_{circ}	T_{area} [min]	$\frac{t_{ext}}{t_{ret}}$
8.94 ± 0.22	26.07 ± 1.70	0.33 ± 0.02	0.84	1.20	1.4
9.14 ± 1.26	55.61 ± 5.18	1.92 ± 0.01	0.86	0.63	1.4
11.66 ± 0.66	20.99 ± 0.47	1.93 ± 0.03	0.63	1.35	2.3
16.72 ± 0.49	25.94 ± 2.48	1.53 ± 0.01	0.84	1.09	1.6
5.73 ± 0.19	63.75 ± 9.75	0.19 ± 0.00	0.92	1.34	1.7
5.96 ± 0.18	81.31 ± 5.08	2.79 ± 0.08	0.84	1.23	1.6
