# Supplementary figures and images for: A lumped parameter model of endoplasm flow in Physarum polycephalum explains migration and polarization-induced asymmetry during the onset of locomotion
Source: PLoS One. 2019 Apr 23;14(4):e0215622. doi: 10.1371/journal.pone.0215622 (PMC6478327; doi:10.1371/journal.pone.0215622)

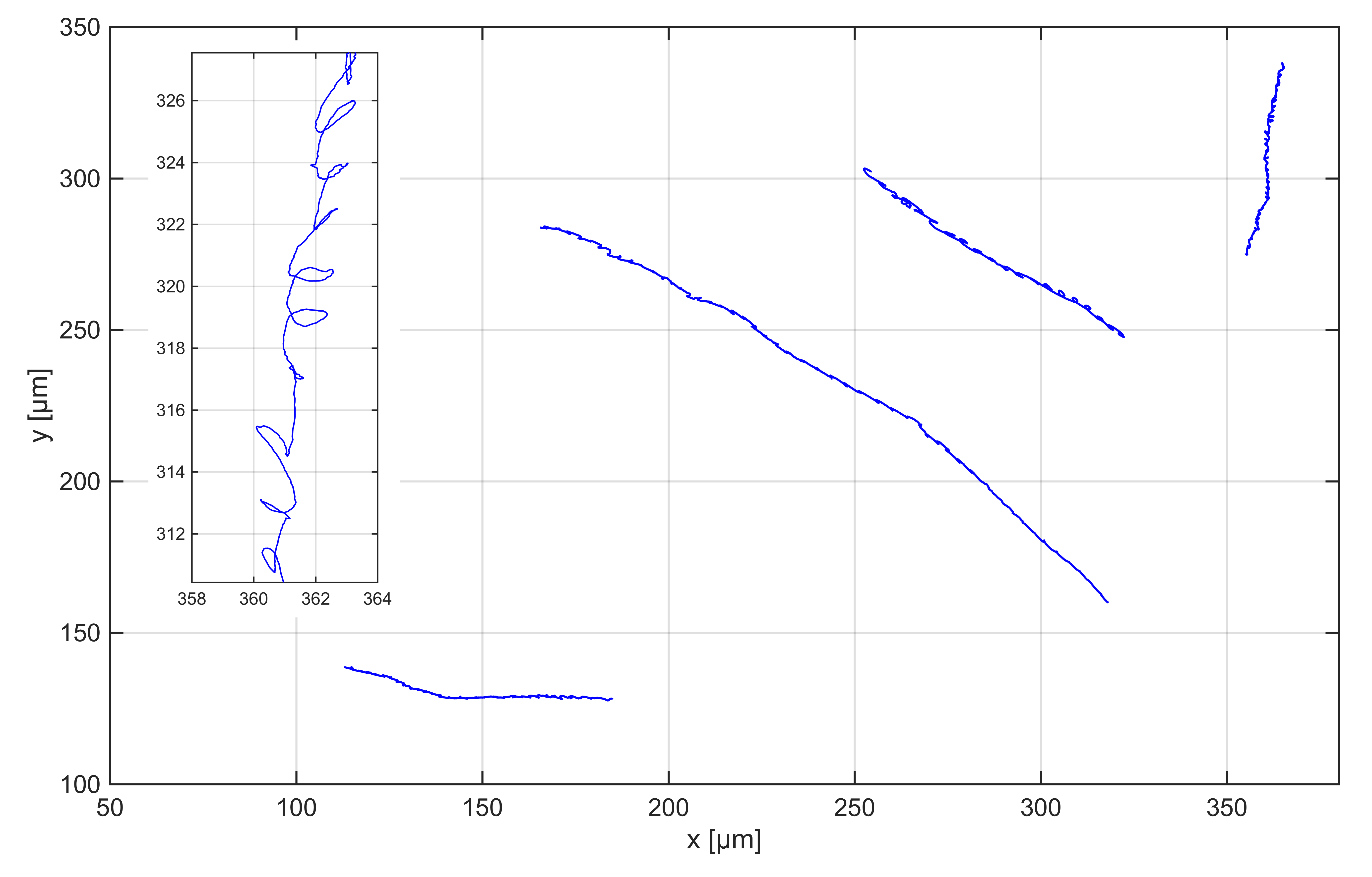

Supplement: S1 Fig — Trajectories of the center of mass of four migrating mesoplasmodia. A straight path is maintained for hours. Inset: Magnification of a detail of a trajectory. The centre of mass of a mesoplasmodium moves along a cycloid path. (TIFF) [file pone.0215622.s004.tiff]

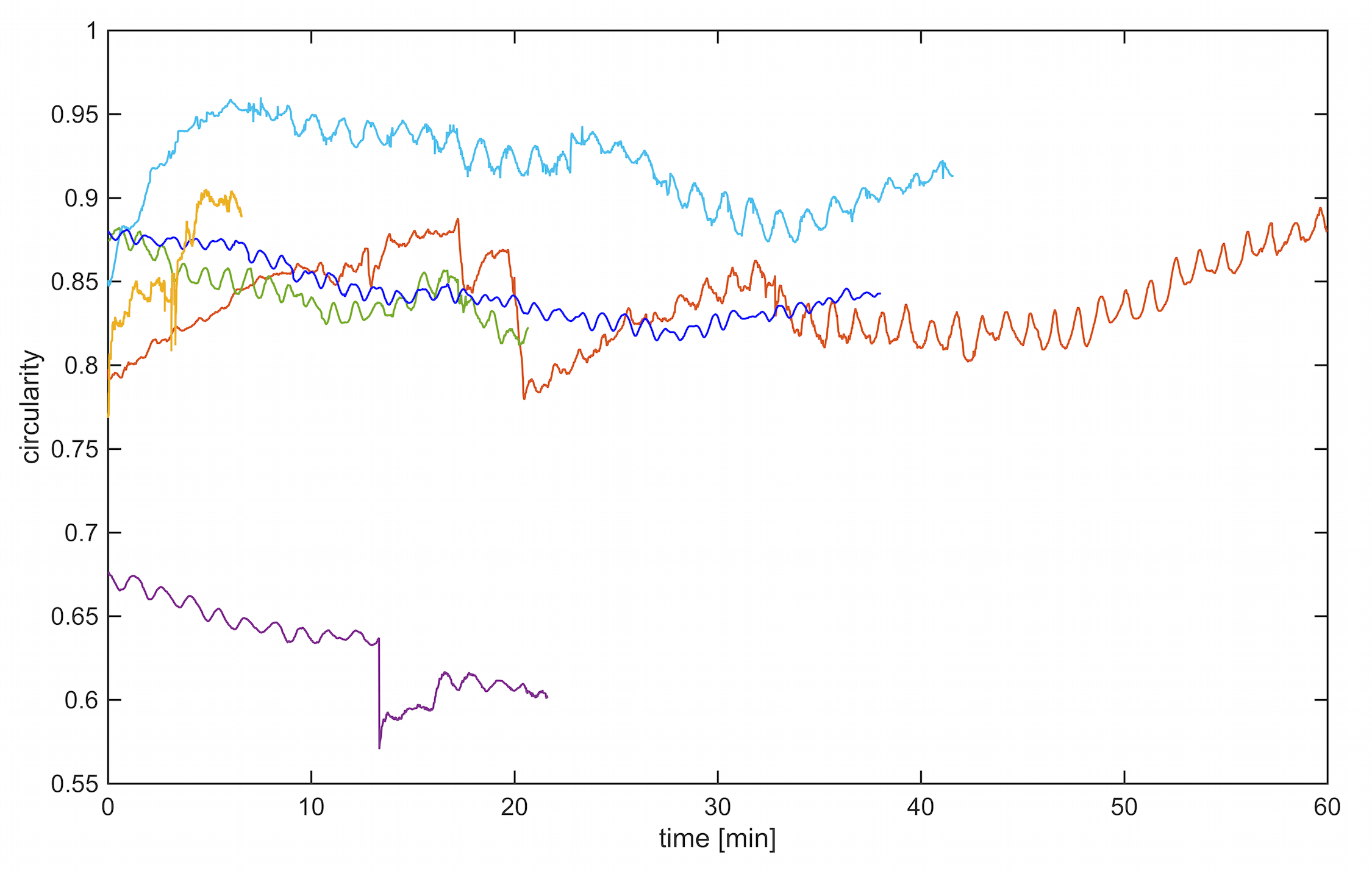

Supplement: S2 Fig — Circularity fcirc over time for six mesoplasmodia. (TIFF) [file pone.0215622.s005.tiff]

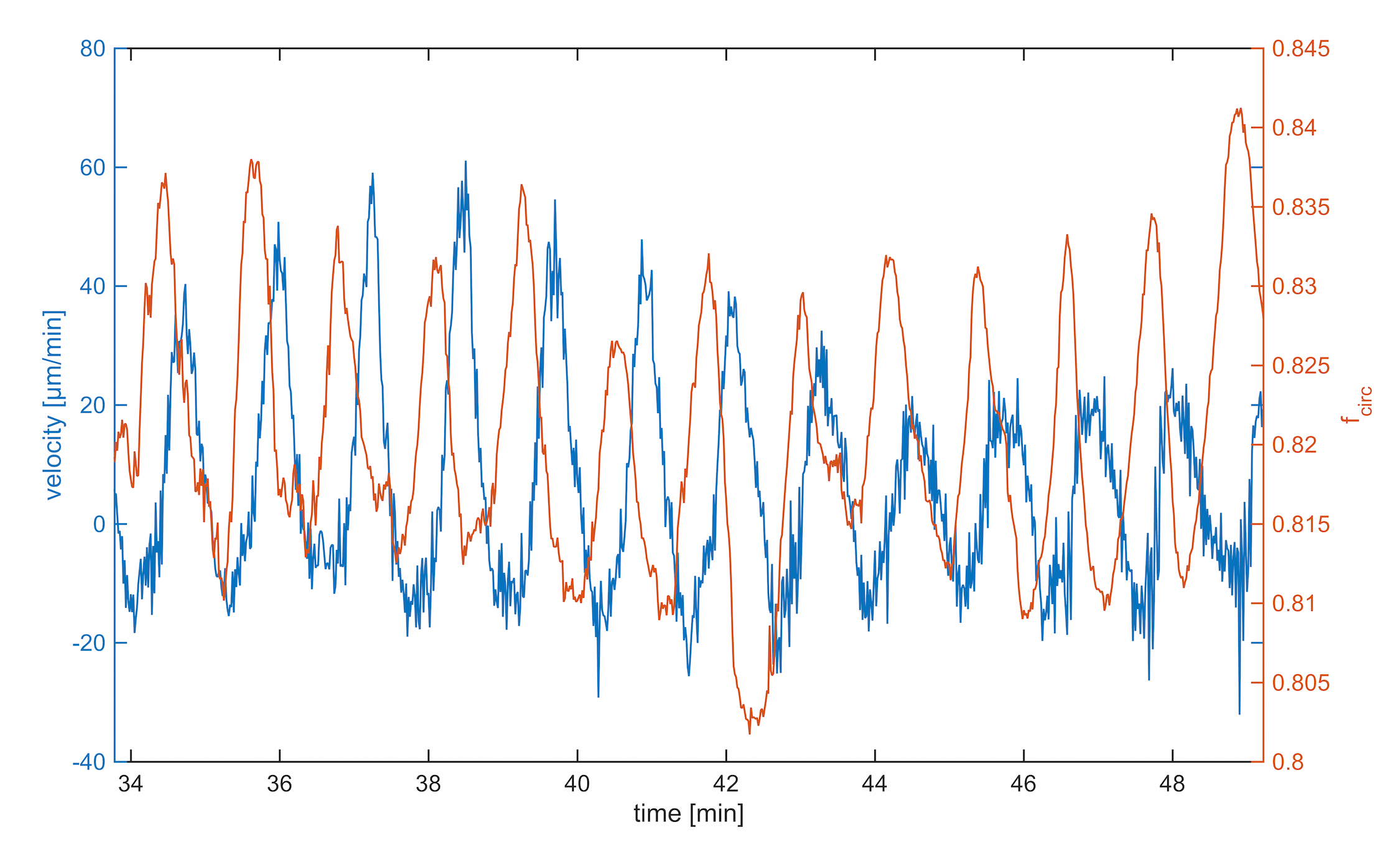

Supplement: S3 Fig — Phases of high circularity correspond to a slowing of locomotion. (TIFF) [file pone.0215622.s006.tiff]
